# Bracelet-like Complexes of Lithium Fluoride with Aromatic Tetraamides, and Their Potential for LiF-Mediated Self-Assembly: A DFT Study

**DOI:** 10.3390/molecules28124812

**Published:** 2023-06-16

**Authors:** Rubén D. Parra

**Affiliations:** Department of Chemistry and Biochemistry, DePaul University, Chicago, IL 60614, USA; rparra1@depaul.edu

**Keywords:** supramolecular chemistry, aromatic tetraamide, LiF-mediated self-assembly, non-covalent interactions, DFT and DFT-D calculations

## Abstract

Geometries and binding energies of complexes between a LiF molecule and a model aromatic tetraamide are obtained using various DFT methods. The tetraamide consists of a benzene ring and four amides positioned so that the LiF molecule can bind via Li⋯O=C or N-H⋯F interactions. The complex with both interactions is the most stable one, followed by the complex with only N-H⋯F interactions. Doubling the size of the former resulted in a complex with a LiF dimer sandwiched between the model tetraamides. In turn, doubling the size of the latter resulted in a more stable tetramer with bracelet-like geometry having the two LiF molecules also sandwiched but far apart from each other. Additionally, all methods show that the energy barrier to transition to the more stable tetramer is small. The self-assembly of the bracelet-like complex mediated by the interactions of adjacent LiF molecules is demonstrated by all computational methods employed.

## 1. Introduction

For more than half a century, supramolecular chemistry has helped expand the scope of traditional molecular chemistry [1,2,3,4]. By extending their attention beyond a single molecule to include an aggregate of molecules, supramolecular scientists have discovered and developed novel materials with potential applications in a variety of fields that could have not been achieved by the individual molecules making up the multimolecular aggregates [5,6,7,8,9,10,11,12,13,14,15,16,17,18,19,20,21,22]. In contrast to the covalent bonds that dominate the structure of a single molecule, non-covalent bonding interactions dominate the assembling of individual molecules into supramolecular aggregates or complexes. Commonly encountered non-covalent bonding include van der Waals, dipole–dipole, ion–dipole, ion–ion, ion–π, π–π stacking, and hydrogen bonding interactions [23,24,25]. The more recent recognition and use of other interactions such as the tetrel, pnicogen, chalcogen, and halogen bonds have enriched the non-covalent bonding toolbox available to supramolecular scientists [26,27,28].

In the development of supramolecular materials, attention is given to the selection or design of individual molecules possessing the type of functionalities that can interact with one another in a complementary manner whenever the molecules are brought together. The specific functionalities in individual molecules would depend on the kind of intermolecular interactions intended to drive the self-assembling of the molecules into the desired supramolecular material. The building block of a supramolecular material can be a single molecule or several identical or different molecules. A group of molecules might aggregate themselves first to form a larger unit that can then be used as a motif for a supramolecular material. An aggregate of molecules can be envisioned with the potential to serve as a host of another molecular system or a metal ion, i.e., host–guest chemistry. Interesting and intriguing possibilities may arise if the hosts themselves can interact with one another via non-covalent interactions to form a large or polymeric array of self-assembled hosts.

In this work, various DFT methods are employed to study the formation of a tetrameric aggregate using lithium fluoride, LiF, and a model benzene tetraamide as basic motifs. One appealing aspect of the amide group is its central role in biological systems, namely, as a repeating unit in polypeptides. Also appealing is the potential for intermolecular self-recognition through hydrogen bonding on account of the amide’s two complementary functional groups, C=O and N-H [29]. This potential for self-assembling has been realized in various ways, including the self-assembling of peptide nanotubes [11]; it has also inspired applications in nanotechnology [30]. Because of its amphiphilic character, the amide group exhibits an ability to bind either electron-deficient species through its C=O group or electron-rich species through its N-H group. Accordingly, research efforts have been devoted to the design of amide-containing ligands for the selective binding of anions or metal cations. Given their potential for π···π and metal ion···π interactions, aromatic amides have been the focus of much attention as well [31]. Examples of macrocyclic aromatic tetraamide receptors for either anions or alkali metal ions have been reported [32,33]. Examples of cooperative binding of ion pairs by amide-containing ligands exist, although these examples usually include the amide motif in conjunction with a different group such as a crown ether [34,35,36]. In a previous publication, the Li^+^ and F^-^ ion-pair binding ability of the same model aromatic tetraamide used in this work was examined computationally [37]. In the work reported here, however, the attention is focused on the ability of the model tetraamide to form stable complexes with the molecule lithium fluoride, LiF. The potential for self-assembly into a tetrameric complex is also examined. The stability of the tetrameric complex is expected to result primarily from the interplay of hydrogen bonding, metal–oxygen, and π–π non-covalent interactions. In addition to its relatively small size, which makes it convenient for computational studies, lithium fluoride is known to form a very stable cyclic dimer [38,39,40,41,42]. Accordingly, the potential for LiF-mediated self-assembly of the tetrameric aggregate itself is investigated. In this case, the tetrameric building blocks are held together via the formation of a LiF-dimer moiety linking adjacent tetramers. The results of this work should have implications in the field of supramolecular chemistry in general. Because of the distinct possibility that the LiF molecule might dissociate into its constituent ions, under the right circumstances, this work should also be of basic importance for the understanding and development of heteroditopic ion-pair receptors [43,44]. Moreover, this work may have some implications in the field of lithium batteries, given the importance of the structural state of lithium compounds in this field [45,46,47]. For example, dual-ion intercalating cell designs, using graphitic anodes and cathodes but employing LiF as the functional salt, dissolved in nonaqueous solvents, have been studied as alternatives to conventional Li-ion cells [45].

## 2. Results and Discussion

### 2.1. LiF–Tetraamide Complexes, mLiF

**Figure 1:**
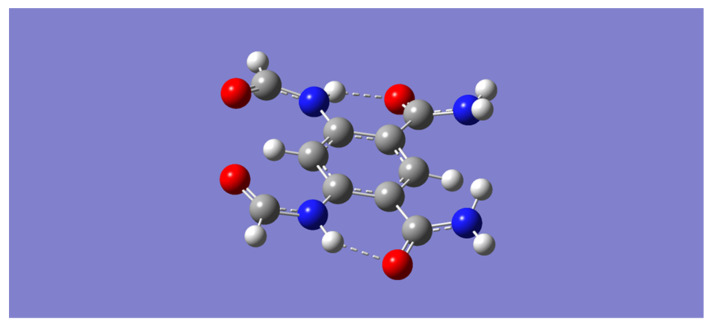
B3LYP/6-31 + G(d) optimized geometry of the model aromatic tetraamide. The color representation is as follows: white (H), gray ©, blue (N), and red (O).

For convenience, the model aromatic tetraamide will be referred to with the letter “m”. All methods give a similar optimized geometry of the model tetraamide, m, as that displayed in Figure 1. Inspection of Figure 1 shows that two of the four amides connect to benzene via their carbonyl carbons, and that the other two amides connect via their nitrogen atoms. In this geometry, the two pairs of amides are connected through N-H⋯O=C hydrogen bonds. In addition, it is apparent in this geometry that the two pairs of amides possess complementary binding ability, which allows for the binding of an electron-rich species through hydrogen bonding with the N-H moieties of one pair of amides, and the binding of an electron-deficient species with the O=C moieties of the other pair of amides [37,43].

**Figure 2:**
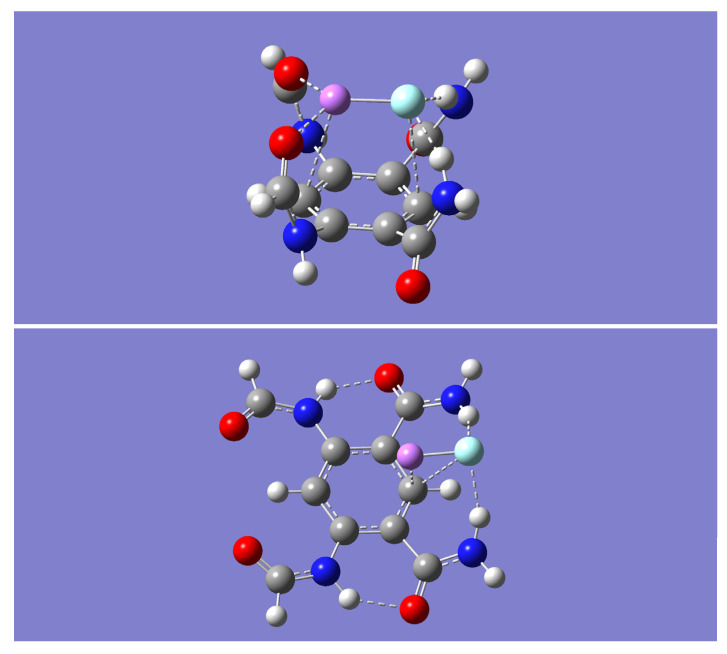

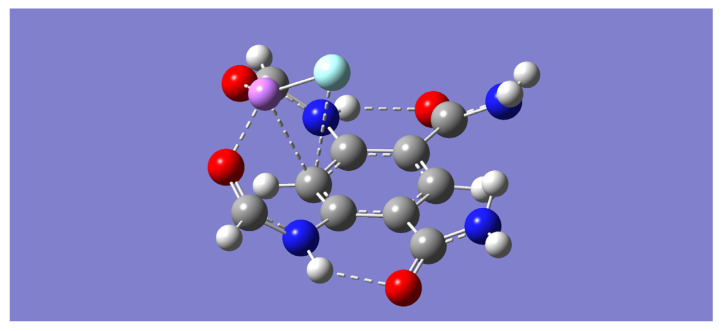
B3LYP/6-31 + G(d) optimized geometries for the dimer complexes of LiF and the model tetraamide, m. Three distinct minimum energy complexes are displayed mLiF-A (**top**), mLiF-B (**middle**), and mLiF-C (**bottom**). The color representation is as follows: white (H), gray (C), blue (N), red (O), pink (Li), and green (F).

The extent of this complementary binding ability is investigated by optimizing the geometry resulting from bringing together the model tetraamide, m, and a molecule of lithium fluoride, LiF, from various intermolecular approaches. Three distinct minimum energy complexes (with zero imaginary frequencies), mLiF-A, mLiF-B, and mLiF-C, were obtained with the B3LYP method (see Figure 2). These optimized dimer complexes were further optimized with the M05-2X method and both the B3LYP-D and the wB97XD methods. In all cases, frequency calculations confirm that the optimized geometries were minima (no imaginary frequencies). The most striking finding is that the mLiF-C complex, obtained with the B3LYP method, is converted into the mLiF-A complex upon geometry optimization with any of the other three methods.

Table 1 shows the relative electronic energies and zero-point energies in kcal/mol for all complexes. All methods predict that mLiF-A is lower in energy relative to mLiF-B. Including the small zero-point energy correction, the smallest relative energy is found with the B3LYP method, 10.91 kcal/mol. All the other methods give corresponding relative energies that are close to one another, with the largest value given by the B3LYP-D method, 14.77 kcal/mol. Interestingly, the mLiF-C complex is only 4.15 kcal/mol above mLiF-A even though it does not remain as a minimum with the other three methods but rather is converted into the mLiF-A complex.

The BSSE-corrected binding energies calculated with the various methods are given in Table 2. The binding energies listed in Table 2 as BE are calculated as the difference between the energy of the complex and the sum of the energies of the constituent monomers, assuming each monomer has the same geometry as in the corresponding complex. All methods agree that the binding energies for all complexes are large and that, except for the wB97XD method, the binding energy of the mLiF-A complex more than doubles that of the mLiF-B complex. The mLiF-A binding energy calculated with the wB97XD method is slightly less than twice that of the mLiF-B complex. Additionally, the B3LYP results show that the binding energy of the mLiF-A complex is more than the sum of the other two complexes, mLiF-B and mLiF-C, found with this method. Relative to the other three methods, B3LYP underestimates the binding energies for the mLiF-A and mLiF-B complexes. Lastly, the M05-2X binding energy of either mLiF-B or mLiF-A lies in between that calculated with the B3LYP-D and wB97XD methods. The binding energies listed in Table 2 as BE-DE take into account the increase in energy of the monomers in the complex due to the geometrical deformations that each monomer suffers upon complex formation. Inspection of Table 2 shows that the energy cost of the geometry deformation of the monomers, relative to their geometries when they are isolated and fully optimized, is much larger upon mLiF-A formation (27.59 to 32.26 kcal/mol) than upon mLiF-B formation (3.29 to 5.24 kcal/mol).

Pertinent geometrical parameters for the mLiF dimers optimized with the various theoretical methods are displayed in Table 3 (distances) and Table 4 (angles). In general, the covalent Li-F bond is elongated relative to the optimized Li-F monomer. For reference, the optimized Li-F bond distance is calculated to be 1.587, 1.583, 1.583, and 1.598 Å with the B3LYP, M05-2X, B3LYP-D, and wB97XD methods, respectively. In particular, the Li-F bond distance is longer in the mLiF-A complex than in the other two complexes. The larger elongation of the Li-F bond in mLiF-A results from the simultaneous chelate binding of Li and F by the aromatic tetraamide. In mLiF-B, the binding is primarily on the F end of the molecule, and in mLiF-C, the binding is on the Li end of the molecule, as revealed in the intermolecular Li⋯O=C and N-H⋯F distances and angles. Additional interactions of the Li-F molecule with the aromatic ring can be inferred from the Li or F distance to the closest carbon in the aromatic ring, as shown in the respective Li⋯C and F⋯C distances in Table 3.

Complexation of LiF in mLiF-A produces a substantial weakening of the intramolecular N-H⋯O=C hydrogen bond, as observed in the respective N-H⋯O distances (Table 3) and angles (Table 4). As a reference, the N-H⋯O distance in the optimized tetraamide monomer, m, is calculated to be 1.822, 1.856, 1.827, and Å with the B3LYP, M05-2X, B3LYP-D, and wB97XD methods, respectively. In the same order of the DFT methods, the optimized N-H⋯O angle in the tetraamide monomer is calculated to be 138.6, 137.1, 138.1 and degrees, respectively. On the other hand, the distortion of the intramolecular N-H⋯O=C hydrogen bond is much smaller upon formation of the mLiF-B or the mLiF-C complexes. For example, the N-H ⋯O angle in mLiF-B is quite close to that in the optimized tetraamide monomer, and thus the distortion of the hydrogen bond is shown mostly in the N-H⋯O distance, which is somewhat lengthened in the complex.

The geometrical flexibility of the model tetraamide to bind LiF is demonstrated in the dihedral angles that relate the two amides forming the intramolecular N-H⋯O=C hydrogen bond with each other in relation to the aromatic ring carbons, namely, the (O=)C-N-C-C and the O=C-C-C dihedral angles listed in Table 5. The values of these dihedral angles reflect the deviation of each amide from the aromatic ring plane. Accordingly, the more aligned the two amides are with the plane, the closer to 180 degrees and to 0 degrees the (O=)C-N-C-C and the O=C-C-C dihedral angles are, respectively. Inspection of Table 5 shows that the amides are aligned with the aromatic plane the most in the uncomplexed tetraamide monomer, m. In contrast, the least alignment with the plane is seen in the mLiF-A complex.

### 2.2. (m-LiF)_2_ Complexes

**Figure 3:**
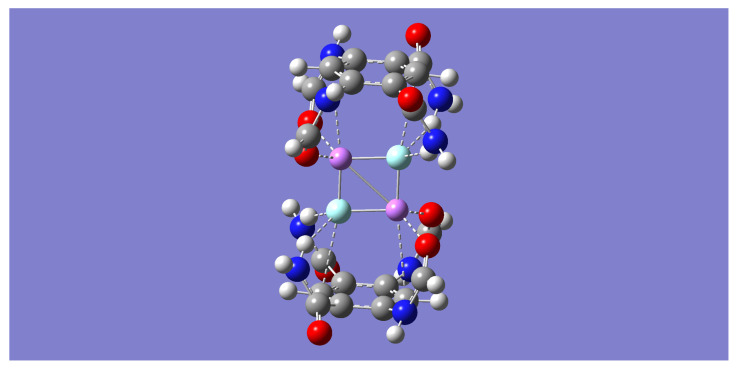
B3LYP/6-31 + G(d) optimized geometry for the (m-LiF)_2_-A tetramer complex. The color representation is as follows: white (H), gray (C), blue (N), red (O), pink (Li), and green (F).

**Figure 4:**
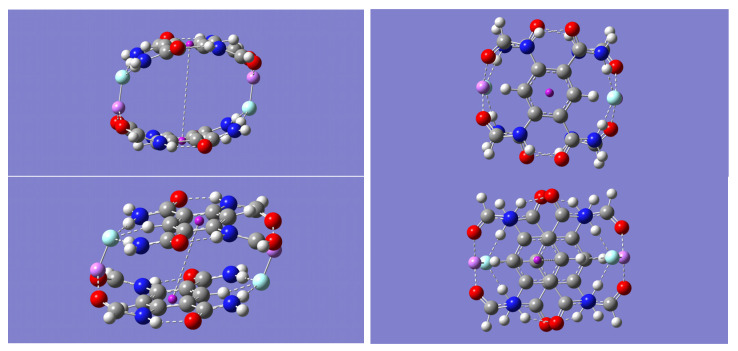
Optimized geometry for the (m-LiF)_2_-B tetramer complex. Upper panel shows the B3LYP/6-31 + G(d) geometries side view (**left**) and top view (**right**). Lower panel shows the M05-2X/6-31 + G(d) geometries side view (**left**) and top view (**right**). The color representation is as follows: white (H), gray (C), blue (N), red (O), pink (Li), and green (F). A purple color is assigned to the dummy atoms placed at the center of each benzene ring.

Each of the dimer complexes, mLiF-A or mLiF-B, was used as a motif to build a dimer of a dimer complex or a tetramer complex, (m-LiF)_2_. Two distinct tetramer complexes, (m-LiF)_2_-A and (m-LiF)_2_-B, were found upon geometry optimization by all the computational methods employed in this work. The former has a C_2_ point group symmetry (Figure 3) while the latter has a bracelet-like geometry of C_2h_ symmetry (Figure 4). The absence of any imaginary frequencies also verified that the optimized tetramers are indeed minima. Specifically, the complementary overlap of two mLiF-A complexes resulted in the (m-LiF)_2_-A tetramer complex, while the corresponding overlap of two mLiF-B complexes resulted in the (m-LiF)_2_-B tetramer. Incidentally, using the optimized ion-pair (Li^+^ and F^−^) complex of the model tetraamide reported previously [37] also resulted in the (m-LiF)_2_-B tetramer.

Table 6 shows the relative electronic and zero-point energies in kcal/mol for all tetramer complexes. All methods predict (m-LiF)_2_-B to be lower (by more than 20 kcal/mol) in energy relative to its (m-LiF)_2_-A counterpart. Including the small zero-point energy correction, the largest relative energy is found with the B3LYP method, which shows (m-LiF)_2_-A above (m-LiF)_2_-B by 27.67 kcal/mol. In turn, the M05-2X gives the smallest energy gap between these complexes, 20.67 kcal/mol. The transition state structure connecting the two tetramers was found by each of the DFT methods employed here. Table 6 shows that the energy barrier, including zero-point energy, for the transition from the higher to the lower energy tetramer is relatively small, with the B3LYP method giving the lowest energy barrier (2.44 kcal/mol).

The BSSE-corrected binding energies for the two tetramers, calculated with the various methods, are given in Table 7. Examination of Table 7 indicates that the B3LYP consistently underestimates the binding energies relative to the other three methods, partly due to the inability of B3LYP to account for dispersion forces such as the π⋯π interactions resulting from the overlap of the two benzene ring units [48,49,50]. The binding energy calculated for the (m-LiF)_2_-B complex corresponds to the breaking of the complex into its four constituent units and is listed as (m-LiF)_2_-B_4 in Table 7. In this case, all methods predict binding energies that are substantially larger than twice the binding energy of the parent dimer mLiF-B. The result is not surprising, given that the Li end of each LiF unit is chelated by amide oxygens in the tetramer, an interaction which is missing in the parent dimer. That is, the much stronger Li⋯O=C interactions in (m-LiF)_2_-B replace the weak Li⋯π interaction in mLiF-B. Regarding the (m-LiF)_2_-A tetramer, two different binding energies were calculated. One shows the binding energy associated with all four units, the two LiF molecules and the two model tetraamides, (m-LiF)_2_-A_4. In this case, all methods predict binding energies that are also more than twice the binding energy of the parent dimer mLiF-A. The difference can be traced down to the interaction between the LiF molecules in the LiF dimer mediating the formation of (m-LiF)_2_-A. Consequently, the (m-LiF)_2_-A complex can be thought of as a dimer of dimers mediated by the interaction of two LiF molecules, and the other binding energy, (m-LiF)_2_-A_2, essentially shows the energy required to break the interaction of the two LiF moieties holding the two dimers together. In this case, the calculated binding energies are similar across the various methods (33.57 to 35.86 kcal/mol). Subtracting this binding energy from that calculated in (m-LiF)_2_-A_4 gives results that are indeed close to twice the binding energies found for the parent dimer mLiF-A (Table 3). Moreover, a comparison of the (m-LiF)_2_-A_2 binding energy can be made with that of the breaking of the optimized LiF dimer into the two constituent LiF monomers. To this effect, the LiF dimer was first optimized yielding a cyclic structure of D_2h_ symmetry, and the corresponding BSSE-corrected binding energy was calculated next. These results agree with previously published results [38,39,40,41,42]. The binding energies shown in Table 7 indicate that the binding energy in the isolated LiF dimer (66.08 to 69.71 kcal/mol) is slightly less than twice that in the (m-LiF)_2_-A_2. That is, the chelate binding, NH⋯F-Li⋯O=C, of each Li-F unit by the amide motifs weakens (by almost 50%) the strength of the interactions in the isolated LiF dimer. Consequently, the binding energy of the LiF dimer in the tetramer is decreased relative to that of the isolated LiF dimer, as demonstrated by all the methods employed.

Relevant geometrical parameters for the (m-LiF)_2_-A tetramer are displayed in Table 8. These parameters can be compared with those for the mLiF-A parent dimer listed in Table 3 (distances) and Table 4 (angles). Table 8 shows two sets of different values for each of the Li-F, Li⋯O=C, and NH⋯F distances in (m-LiF)_2_-A, which are in all cases longer than the corresponding values in Table 3, regardless of the method used. The larger Li-F bond distances correspond to those of the LiF units in the same geometrical orientation as that in mLiF-A, while the smaller distances correspond to the separation of the adjacent LiF molecules holding together the (m-LiF)_2_-A tetramer. For additional comparison, the smaller Li-F distances are still larger than those found in the isolated LiF dimer of D_2h_ symmetry. Specifically, the Li-F distance in the LiF dimer obtained by the B3LYP, B3LYP-D, M05-2X, and wB97XD methods are 1.735, 1.737, 1.726, and 1.745 Å, respectively. Two other parameters listed in Table 8 are the symmetric distances from each Li or F to its closest aromatic carbon, Li⋯C or F⋯C, respectively. The former shows an elongation relative to that in the parent dimer, while the opposite is true for the latter. Lastly, the Li⋯O=C and N-H⋯F angles change slightly upon tetramer formation. For example, the B3LYP method shows a widening of about 3.6 degrees for the Li⋯O=C angles concomitant with a narrowing of 3.6 degrees for the N-H⋯F angles. The widening for the Li⋯O=C angles is smaller with the other three methods, with a slightly larger N-H⋯F narrowing.

Important geometrical parameters for the bracelet-like (m-LiF)_2_-B tetramer are displayed in Table 9. Comparable parameters for the mLiF-B parent dimer are listed in Table 3 (distances) and Table 4 (angles). In particular, the binding of each Li end of a LiF molecule by the oxygen atoms of the pertinent amides in each aromatic ring in the tetramer results in a substantial decrease in the Li⋯O=C distances relative to those in the parent dimer where these interactions are missing. The Li⋯O=C angles are in tandem widened greatly upon tetramer formation. Similar results are found for the N-H⋯F hydrogen bond distances and angles, indicating stronger hydrogen bond interactions in the tetramer. Even the intramolecular N-H⋯O=C hydrogen bond is strengthened in the tetramer, as demonstrated mostly by the sizeable decrease in the pertinent hydrogen bond distance. One striking interaction that is absent in the parent mLiF-B dimer but that emerges in the (m-LiF)_2_-B tetramer is the intermolecular C-H⋯F hydrogen bond interaction. Except for the B3LYP method, all other methods consistently show relatively small hydrogen bond distances (1.842–1.863 Å) and quasilinear angles (168.2–172.6 degrees). The B3LYP method, however, shows a much larger C-H⋯F hydrogen bond distance (2.132 Å) and narrower angle (127.6 degrees), suggesting that the C-H⋯F hydrogen bond interactions make negligible contributions to the stability of the tetramer at the B3LYP level. Another interaction that is unique to the (m-LiF)_2_-B tetramer complex is the π⋯π interaction resulting from the overlapping of the two aromatic rings. Table 9 shows the distance separating the centers of each aromatic ring. Except for the B3LYP method, all the other methods predict relatively small distances between the aromatic rings (3.852–4.092 Å). The much larger distance between the aromatic ring centers predicted by the B3LYP method, 5.378 Å, is consistent with the inability of this method to account for dispersion forces, and thus any contribution to the stability of the tetramer due to the π⋯π interaction is essentially unaccounted for with the B3LYP method. It is worth noting that the B3LYP method predicts an almost perfectly eclipsed orientation of the two aromatic rings, while the other three methods predict a staggered orientation with the center of one ring directly above one of the carbon atoms in the other ring (see Figure 3). Although not shown in Table 9, the intermolecular Li⋯F separation is close to 7 Å with the B3LYP method and close to 8 Å with the other three methods.

### 2.3. Self-Assembly: [(m-LiF)_2_-B]_n_ Complexes

**Figure 5:**
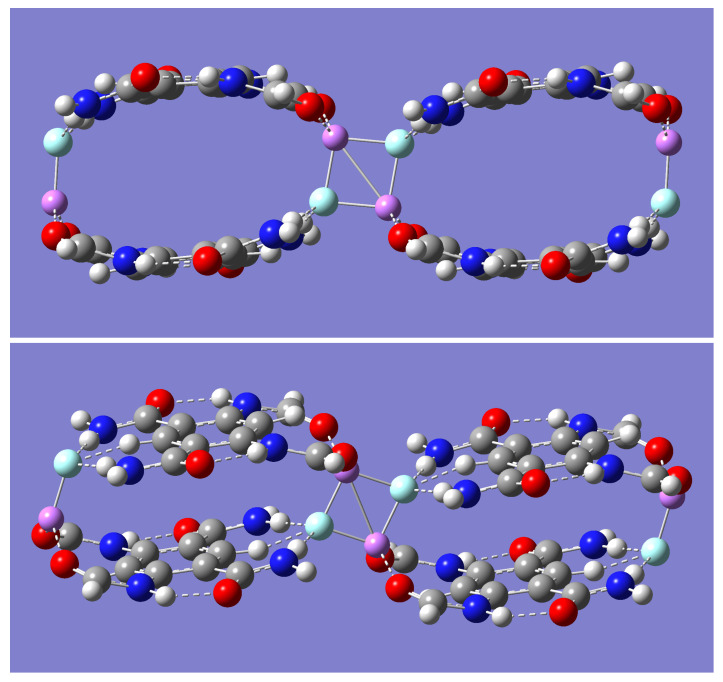
Optimized geometry for the [(m-LiF)_2_]_2_-B self-assembled complex. **Upper** panel shows the B3LYP/6-31 + G(d) geometry. **Lower** panel shows the M05-2X/6-31 + G(d) geometry. The color representation is as follows: white (H), gray (C), blue (N), red (O), pink (Li), and green (F).

Although both (m-LiF)_2_-A and (m-LiF)_2_-B tetramer complexes exhibit large binding energies, it is the latter complex that offers a compelling possibility for self-assembly into a growing network of *n* ≥ 1 tetrameric units, [(m-LiF)_2_-B)]_n_. Indeed, the potential for the interaction of adjacent tetrameric units via their LiF units seems plausible, as was seen in the formation of the (m-LiF)_2_-A complex from two mLiF-A parent dimers. In the (m-LiF)_2_-A complex, however, continuous self-assembly via the LiF dimer interaction is not possible. In the case of the (m-LiF)_2_-B self-assembly, the possibility for continuous network growth remains after two or more tetrameric units are brought together. This possibility was explored first for the case of *n* = 2. Geometry optimization and frequency calculations with the B3LYP method resulted in a [(m-LiF)_2_-B)]_2_ complex of D_2h_ symmetry (See Figure 5) whose nature as a minimum energy structure was confirmed by the absence of any imaginary frequencies. In this larger complex, each tetrameric unit is linked sideways with an adjacent tetramer in an interaction mediated by the formation of the LiF dimer in a way that resembles the D_2h_ symmetry of the isolated LiF dimer, as it did in the (m-LiF)_2_-A complex. Similar results were also found with the other three methods.

The energy required to break the [(m-LiF)_2_-B)]_2_ complex into its two tetrameric units was calculated and the results are listed in Table 10. This energy can be appropriately compared with the energy required to break the (m-LiF)_2_-A complex into its constituent dimers, (m-LiF)_2_-A_2 (Table 7). In general, the calculated BSSE-corrected binding energies are just few kcal/mol smaller than those listed in Table 7. More specifically, the largest decrease in the [(m-LiF)_2_-B)]_2_ complex binding energies occurs with the B3LYP method (−16%), and the smallest decrease occurs with the wB97XD method (−6%). The corresponding decreases with the other two methods B3LYP-D (−8%) and M05-2X (−9%) are close to each other. Despite the noted decrease relative to (m-LiF)_2_-A_2, the calculated binding energies in the [(m-LiF)_2_-B)]_2_ complex are still large enough (in the range of 28.04 to 35.90 kcal/mol) to hold the two tetrameric units together.

**Figure 6:**
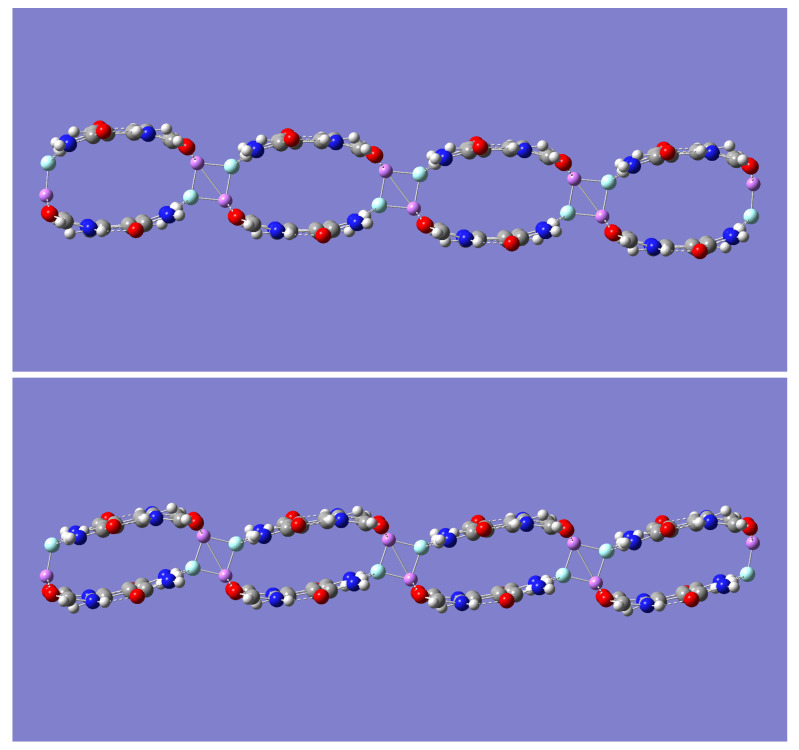
Optimized geometry for the [(m-LiF)_2_]_4_-B self-assembled complex. **Upper** panel shows the B3LYP/6-31 + G(d) geometry. **Lower** panel shows the M05-2X/6-31 + G(d) geometry. The color representation is as follows: white (H), gray (C), blue (N), red (O), pink (Li), and green (F).

To further examine the LiF-mediated self-assembly of the (m-LiF)_2_-B complex, a geometry optimization of the much larger [(m-LiF)_2_-B)]_4_ complex was carried out with the less computationally demanding B3LYP and M05-2X methods. The optimized geometries are shown in Figure 6. The energy required to break the self-assembled complex into its four tetrameric units was calculated as 85.59 and 97.51 kcal/mol with the B3LYP and M05-2X methods, respectively. After dividing each of these binding energies by the number of mediating LiF dimers holding the [(m-LiF)_2_-B)]_4_ complex, the results (28.53 and 32.50 kcal/mol) are essentially the same as those obtained for the [(m-LiF)_2_-B)]_2_ complex with each of these methods. Moreover, the energy required to break the [(m-LiF)_2_-B)]_4_ into two complexes of size *n* = 3 and *n* = 1 is 32.50 kcal/mol, while that to break it into two equivalent complexes of size *n* = 2 is 32.65 kcal/mol with the M05-2X method. The corresponding results with the B3LYP method are, respectively, 28.47 and 28.85 kcal/mol. The results are important as they suggest that the binding energy for the LiF-mediated self-assembly in [(m-LiF)_2_-B)]_n_ is independent of its size, *n*.

Inspection of Figure 4 reveals one striking geometrical distinction between the relative orientation of the tetrameric units, upon self-assembly, predicted by the B3LYP and M05-2X methods. The latter method predicts a geometry with the adjacent tetrameric units linked in a staircase manner, while the former method maintains the tetrameric units linked along the same line, one each for the top and the low end of the complex. Relevant geometric data are presented in Table 11 (distances) and Table 12 (angles) for both [(m-LiF)_2_-B)]_2_ and [(m-LiF)_2_-B)]_4_. Table 11 lists two different sets of Li-F distances. One set refers to the distance within each of the Li-F monomers already present in the parent (m-LiF)_2_-B parent (the outer LiF molecules), and the other set refers to the distance between the adjacent LiF molecules (the inner LiF molecules), one from each (m-LiF)_2_-B parent, involved in the LiF-mediated self-assembly. For [(m-LiF)_2_-B)]_2_, cross-inspection of Table 9 and Table 11 shows that all methods predict the Li-F distance for each of the outer LiF monomer to remain essentially unchanged when compared with the value it has in the parent tetrameric unit. In contrast, the Li-F distance for each of the inner LiF monomers is significantly lengthened, with an Li-F distance that is closer to that between the LiF molecules in the mediating LiF dimer. The trends noted for the outer and inner Li-F distances are generally true for the other distances listed in Table 11, Li⋯O=C, N-H⋯F, and C-H⋯F. One exception is the inner C-H⋯F distance for which the B3LYP method predicts an inner distance that is shorter than the outer one. Lastly, the distance between the centers of the aromatic rings decreases slightly in [(m-LiF)_2_-B)]_2_ relative to that in (m-LiF)_2_-B. With respect to the angles, Table 12 shows that while the outer Li⋯O=C, N-H⋯F, and C-H⋯F angles remain very close to their original values in the parent tetramer, the inner angles exhibit a small increase. It should be noted that the B3LYP method, in contrast to all other methods, predicts a sizeable increase for the inner C-H⋯F angles. Regarding the larger [(m-LiF)_2_-B)]_4_ self-assembled complex, Table 11 shows that the listed outer and inner distances remain close to their values in [(m-LiF)_2_-B)]_2_. Interestingly, the distance between the centers of the inner aromatic rings still shows a slight decrease in [(m-LiF)_2_-B)]_4_. The trends in the Li⋯O=C, N-H⋯F, and C-H⋯F angles also mirror those found in [(m-LiF)_2_-B)]_2_.

## 3. Computational Methods

All calculations were performed using the Gaussian 16 program [51]. Four different density functional methods were used for geometry optimization and frequency calculations. The B3LYP and M05-2X functionals were used along with the 6-31 + G(d) basis set. The B3LYP-D and wB97XD functionals were used along with the larger basis set 6-311 + G(d,p). Calculated binding energies for all complexes were corrected for basis set superposition error (BSSE) [52]. Please note that optimized geometries are provided as Supplementary Material.

It is worth noting that the B3LYP functional consists of the Becke exchange functional (B), the LYP non-local correlation functional, and the VWN functional III for local correlation [53,54,55,56]. Historically, B3LYP has been one of the most used functional methods for geometry optimization of molecules and the study of complexes including those exhibiting hydrogen bonding interactions [57,58]. Despite its popularity, especially in the 1990s and at the beginning of the 21st century, B3LYP has been shown to have serious limitations, particularly in those cases where dispersion interactions are important [48,49,50]. Using a DFT method that includes dispersion corrections is, then, generally desired [59,60,61]. Both the B3LYP-D and the wB97XD functionals include the empirical Grimme’s D3 and D2 dispersion methods, respectively [62,63,64,65]. The wB97XD functional also includes long-range corrections [66]. It is important to note, nonetheless, that using the wB97XD functional for accurate modeling of orbital energy in conjugated molecules has been found to depend on the range-separation parameter (ω). This parameter’s optimal value is not always known and must be tuned since the default value for ω overestimates HOMO/LUMO and optical gaps when compared to experimental values [67]. Furthermore, on a benchmark of DFT methods on halogen bonds, it was found that, for B3LYP, for example, the addition of dispersion corrections might actually degrade binding-energy accuracy [68]. Lastly, the M05-2X functional, which accounts for medium-range electron correlation, has been shown to perform well for non-covalent interactions, especially weak interaction, hydrogen bonding, and π···π stacking [69,70,71,72]. Some studies show, however, that because of the neglect of long-range correlation in the M05-2X, the performance of this functional in describing hydrogen-bonded nucleic acid base pairs is generally inferior to that of functionals including dispersion corrections [73]. Moreover, some authors recommend employing DFT-D functionals rather than the M05-2X for complexes with π···π interactions because the M05-2X tends to underestimate severely the interaction energy [74]. As shown in the results section below, the complexes investigated in this work exhibit an interplay of various interactions, including intramolecular N-H⋯O=C hydrogen bonding, relatively strong intermolecular N-H⋯F and weak C-H⋯F hydrogen bonding, Li⋯O=C binding, and weak π⋯π interactions. Thus, the use of the different DFT methods in this work helps determine whether a consistent set of results can be obtained regarding the geometry and stability of the systems under investigation. For convenience, in this work the combination of the density functional method and the chosen basis set will be referred to by using just the functional. For example, the B3LYP method implies here B3LYP/6-31 + G(d), and so on.

## 4. Future Perspectives

Four different density functional theory methods were used to study the geometries and binding energies of dimer complexes between a model aromatic tetraamide and a molecule of lithium fluoride. In two of the methods, B3LYP and M05-2X, the 6-31 + G(d) basis set was used; in the other two methods, B3LYP-D and wB97XD, the larger 6-311 + G(d,p) basis set was used instead. Three dimer complexes were found with the B3LYP method. In the mLiF-A complex, the aromatic tetraamide binds the LiF molecule via chelate binding of both the Li atom and the F atom. The former atom interacts with the oxygen atoms of the amide groups on one end, the C=O moieties, and the latter atom forms hydrogen bonding with N-H moieties of the amide groups on the other end. In the other two dimer complexes found by the B3LYP method, the binding of the LiF molecules occurs either through N-H⋯F hydrogen bonding, mLiF-B, or Li⋯O=C chelate binding, mLiF-C. All the other three methods found that the mLiF-C complex of the B3LYP method does not remain as such, but rather is converted readily into the mLiF-A complex upon geometry optimization. Moreover, all methods agree that the mLiF-A complex has the larger binding energy and is the more stable one. For example, mLiF-A is 14.13 kcal/mol more stable than mLiF-B with the M05-2X. With this method, the binding energy for mLiF-A (46.28 kcal/mol) is about 1.6 times that of mLiF-B (29.69 kcal/mol) after correction for deformation energies.

The possibility to form a dimer of dimers was explored. The results from all computational methods consistently show two distinct tetrameric complexes, (m-LiF)_2_-A and(m-LiF)_2_-B. The former complex is brought about by the interaction of the two LiF molecules in adjacent mLiF-A dimers. Here, the resulting LiF dimer is sandwiched between the aromatic tetraamides. The latter complex adopts a bracelet-like geometry with the two LiF molecules far apart from each other and yet sandwiched by the aromatic amides. The (m-LiF)_2_-B complex is found to be the more stable complex. Additionally, all methods show that the energy barrier to transition from (m-LiF)_2_-A to (m-LiF)_2_-B is small (2.4 to 4.3 kcal/mol).

In addition to being the more stable tetrameric complex, (m-LiF)_2_-B exhibits the potential for LiF-mediated self-assembly, [(m-LiF)_2_-B)]_n_, formed by the interactions of LiF molecules in adjacent (m-LiF)_2_-B units. The realization of such potential is consistently predicted by all computational methods employed in this work. Specifically, a self-assembled complex of size *n* = 2 was found with robust binding energies (between 28 and 36 kcal/mol, depending on the computational method). The B3LYP and M05-2X methods were used to study the much larger self-assembled complex of size *n* = 4. The results show average binding energies that are essentially the same as those in the smaller complex, *n* = 2. Similarly, only minor geometrical changes are seen upon increasing the size of the self-assembled complex.

Further computational and experimental work should be carried out to assess the generality of the results presented in this work and to gain additional insight into the intermolecular forces driving the formation of the complexes studied [75,76]. The author is currently investigating the ability of the bracelet-like complexes to host small molecules or metal ions. Indeed, the self-assembling of the tetrameric hosts should inspire pertinent research in the field of supramolecular chemistry [77,78,79]. In the author’s lab, for example, the generality of the results is being explored computationally using MX motifs to mediate the self-assembly process with different alkali metals, M, or halides, X. The use of metal oxides, such as MgO, is being considered as well. Moreover, the effect of extending the size beyond the benzene ring for the backbone containing the four amides is likewise being investigated in the author’s lab.

## Figures and Tables

**Table 1:** Relative (kcal/mol) electronic energies, ∆E, and zero-point vibrational energies, ∆zpve, of the mLiF complexes.

	**B3LYP**		
**Complex**		**∆** **E**	**∆** **zpve**
mLiF-A		0.00	0.00
mLiF-B		10.70	0.21
mLiF-C		4.05	0.10
	**B3LYP-D**		
**Complex**		**∆** **E**	**∆** **zpve**
mLiF-A		0.00	0.00
mLiF-B		15.19	−0.41
	**M05-2X**		
**Complex**		**∆** **E**	**∆** **zpve**
mLiF-A		0.00	0.00
mLiF-B		13.73	0.40
	**wB97XD**		
**Complex**		**∆** **E**	**∆** **zpve**
mLiF-A		0.00	0.00
mLiF-B		13.30	0.06

**Table 2:** BSSE-corrected binding energies, before (BE) and after correction for deformation energies (BE-DE), of the mLiF complexes *. All values in kcal/mol.

		**BE**		
**Complex**	**B3LYP**	**B3LYPD**	**M05-2X**	**wB97XD**
mLiF-A	64.58	70.99	74.47	71.25
mLiF-B	25.91	29.61	34.66	36.22
		**BE-DE**		
**Complex**	**B3LYP**	**B3LYPD**	**M05-2X**	**wB97XD**
mLiF-A	32.32	40.76	46.28	43.66
mLiF-B	22.13	26.32	29.69	30.98

* MLiF-C is found only with the B3LYP/6-31 + G(d) method with binding and deformation energies of 36.54 and 8.03 kcal/mol, respectively.

**Table 3:** Pertinent geometrical distances (Å) for the various mLiF complexes.

			**mLiF-A Complex**			
**Method**	**Li-F**	**Li** **⋯** **O=C**	**N-H** **⋯** **F**	**N-H** **⋯** **O**	**F** **⋯** **C**	**Li** **⋯** **C**
B3LYP	1.746	2.017	1.860	2.614	2.956	2.917
B3LYP-D	1.752	2.005	1.828	2.546	2.911	2.973
M05-2X	1.736	2.002	1.838	2.481	2.840	2.832
wB97XD	1.771	2.055	1.797	2.581	2.870	2.835
			**mLiF-B Complex**			
**Method**	**Li-F**	**Li** **⋯** **O=C**	**N-H** **⋯** **F**	**N-H** **⋯** **O**	**F** **⋯** **C**	**Li** **⋯** **C**
B3LYP	1.662	5.273	1.918	1.869	2.783	2.315
B3LYP-D	1.664	5.319	1.914	1.867	2.766	2.386
M05-2X	1.669	4.803	1.885	1.913	2.709	2.311
wB97XD	1.696	4.880	1.863	1.902	2.751	2.396
			**mLiF-C Complex**			
**Method**	**Li-F**	**Li** **⋯** **O=C**	**N-H** **⋯** **F**	**N-H** **⋯** **O**	**F** **⋯** **C**	**Li** **⋯** **C**
B3LYP	1.664	2.032	4.522	1.947	3.108	2.641

**Table 4:** Pertinent geometrical angles (degrees) for the various mLiF complexes.

		**mLiF-A Complex**	
**Method**	**Li** **⋯** **O=C**	**N-H** **⋯** **F**	**N-H** **⋯** **O**
B3LYP	134.9	161.1	106.0
B3LYP-D	137.1	160.6	107.1
M05-2X	134.2	159.3	108.2
wB97XD	133.2	161.5	106.1
		**mLiF-B Complex**	
**Method**	**Li** **⋯** **O=C**	**N-H** **⋯** **F**	**N-H** **⋯** **O**
B3LYP	71.6	159.4	138.5
B3LYP-D	72.6	159.4	137.9
M05-2X	75.8	157.9	136.6
wB97XD	75.7	159.9	136.9
		**mLiF-C Complex**	
**Method**	**Li** **⋯** **O=C**	**N-H** **⋯** **F**	**N-H** **⋯** **O**
B3LYP	129.2	109.8	130.5

**Table 5:** Dihedral angles (degrees) relating the amide substituents in the tetraamide monomer, m, and in the various mLiF complexes.

	Free Tetraamide, m	
**Method**	**(O=)C-N-C-C**	**O=C-C-C-C**
B3LYP	174.1	18.9
B3LYP-D	172.4	20.4
M05-2X	172.1	23.2
wB97XD		
	**mLiF-A Complex**	
**Method**	**(O=)C-N-C-C**	**O=C-C-C-C**
B3LYP	98.5	64.8
B3LYP-D	96.8	63.4
M05-2X	97.4	61.6
wB97XD	97.3	64.6
	**mLiF-B Complex**	
**Method**	**(O=)C-N-C-C**	**O=C-C-C-C**
B3LYP	171.7	30.4
B3LYP-D	169.8	30.8
M05-2X	167.4	37.4
wB97XD	166.8	37.4
	**mLiF-C Complex**	
**Method**	**(O=)C-N-C-C**	**O=C-C-C-C**
B3LYP	138.2	27.9

**Table 6:** Relative (kcal/mol) electronic energies, ∆E, and zero-point vibrational energies, ∆zpve, of the (m-LiF)_2_ complexes and connecting transition states, TS.

	**B3LYP**		
**Complex**		**∆** **E**	**∆** **zpve**
(m-LiF)_2_-A		0.00	0.00
(m-LiF)_2_-B		−30.54	2.87
TS		2.59	−0.15
	**B3LYP-D**		
**Complex**		**∆** **E**	**∆** **zpve**
(m-LiF)_2_-A		0.00	0.00
(m-LiF)_2_-B		−27.11	2.02
TS		3.58	−0.25
	**M05-2X**		
**Complex**		**∆** **E**	**∆** **zpve**
(m-LiF)_2_-A		0.00	0.00
(m-LiF)_2_-B		−22.65	1.98
TS		4.64	−0.35
	**wB97XD**		
**Complex**		**∆** **E**	**∆** **zpve**
(m-LiF)_2_-A		0.00	0.00
(m-LiF)_2_-B		−27.36	3.07
TS		4.27	−0.09

**Table 7:** BSSE-corrected binding energies, ∆E (kcal/mol), of the (m-LiF)_2_ complexes and the LiF dimer.

Complex	B3LYP	B3LYP-D	M05-2X	wB97XD
(m-LiF)_2_-B_4	142.45	161.72	160.29	164.14
(m-LiF)_2_-A_4	162.80	181.47	179.70	177.17
(m-LiF)_2_-A_2	33.57	39.10	35.86	35.18
(LiF)_2_	66.08	66.51	69.71	67.02

**Table 8:** Pertinent geometrical distances (Å) and angles (degrees) for the tetrameric (m-LiF)_2_-A complex.

	**Distances**				
**(m-LiF)_2_-A**	**Li-F**	**Li** **⋯** **O=C**	**N-H** **⋯** **F**	**F** **⋯** **C**	**Li** **⋯** **C**
B3LYP	1.909	2.144	1.980	2.935	3.112
	1.802	2.143	1.975	2.935	3.112
B3LYP-D	1.892	2.143	1.958	2.852	3.081
	1.796	2.067	1.886	2.852	3.081
M05-2X	1.871	2.113	1.927	2.780	2.941
	1.789	2.093	1.919	2.780	2.941
wB97XD	1.901	2.192	1.882	2.808	2.944
	1.816	2.145	1.864	2.808	2.944
	**Angles**				
**(m-LiF)_2_-A**	**Li** **⋯** **O=C**	**N-H** **⋯** **F**			
B3LYP	138.5	157.4			
	138.5	157.5			
B3LYP-D	138.4	155.9			
	139.4	139.4			
M05-2X	135.7	154.8			
	135.9	154.8			
wB97XD	134.4	157.6			
	135.0	157.8			

**Table 9:** Pertinent geometrical distances (Å) and angles (degrees) for the tetrameric (m-LiF)_2_-B complex.

			**Distances**			
**(m-LiF)_2_-B**	**Li-F**	**Li** **⋯** **O=C**	**N-H** **⋯** **F**	**C-H** **⋯** **F**	**N-H** **⋯** **O**	**Centers**
B3LYP	1.719	1.968	1.785	2.132	1.841	5.378
B3LYP-D	1.722	1.956	1.807	1.863	1.777	3.906
M05-2X	1.706	1.941	1.800	1.866	1.801	4.092
wB97XD	1.737	1.983	1.787	1.842	1.765	3.852
			**Angles**			
**(m-LiF)_2_-B**	**Li** **⋯** **O=C**	**N-H** **⋯** **F**	**C-H** **⋯** **F**	**N-H** **⋯** **O**		
B3LYP	144.0	175.6	127.6	135.7		
B3LYP-D	143.0	174.3	169.1	138.0		
M05-2X	139.6	176.7	168.2	136.5		
wB97XD	139.7	175.5	172.6	138.3		

**Table 10:** BSSE-corrected binding energies, ∆E, of the tetrameric[m-LiF)_2_-B]_2_ complex.

Method	∆E (kcal/mol)
B3LYP	28.04
B3LYP-D	35.90
M05-2X	32.50
wB97XD	32.95

**Table 11:** Pertinent geometrical distances (Å) for both the [(m-LiF)_2_-B]_2_ complex and the larger [(m-LiF)_2_-B]_4_ complex.

			**[(m-LiF)_2_-B]_2_**			
**Method**	**Li-F**	**Li-F**	**Li** **⋯** **O=C**	**N-H** **⋯** **F**	**C-H** **⋯** **F**	**Centers**
B3LYP	1.717	1.836	1.969	1.787	2.130	5.060
	1.844	1.836	2.050	1.847	2.082	5.060
B3LYP-D	1.722	1.810	1.957	1.805	1.868	3.865
	1.837	1.810	2.009	1.826	1.884	3.865
M05-2X	1.707	1.806	1.941	1.795	1.872	3.974
	1.817	1.806	1.998	1.827	1.906	3.974
wB97XD	1.735	1.829	1.984	1.783	1.849	3.810
	1.840	1.829	2.035	1.810	1.872	3.810
			**[(m-LiF)_2_-B]_4_**			
**Method**	**Li-F**	**Li-F**	**Li** **⋯** **O=C**	**N-H** **⋯** **F**	**C-H** **⋯** **F**	**Centers**
B3LYP	1.716	1.833	1.970	1.787	2.134	5.064
	1.848	1.835	2.050	1.847	2.083	4.831
	1.840	1.832	2.053	1.843	2.110	4.831
	1.844	1.832	2.053	1.844	2.108	5.064
M05-2X	1.706	1.807	1.940	1.794	1.875	3.979
	1.816	1.807	1.998	1.826	1.902	3.913
	1.812	1.806	1.999	1.821	1.913	3.913
	1.814	1.806	1.999	1.820	1.909	3.979

**Table 12:** Pertinent geometrical angles (degrees) for both the [(m-LiF)2-B]2 complex and the larger [(m-LiF)2-B]4 complex.

		**[(M-LiF)_2_-B]_2_**	
**Method**	**Li** **⋯** **O=C**	**N-H** **⋯** **F**	**C-H** **⋯** **F**
B3LYP	144.8	175.4	128.3
	153.9	177.0	139.6
B3LYP-D	143.3	173.8	170.1
	147.6	176.4	166.6
M05-2X	141.4	175.8	169.2
	146.7	177.8	164.4
wB97XD	140.4	175.1	172.6
	145.5	177.6	168.4
		**[(M-LiF)_2_-B]_4_**	
**Method**	**Li** **⋯** **O=C**	**N-H** **⋯** **F**	**C-H** **⋯** **F**
B3LYP	145.0	175.4	127.9
	154.2	177.0	139.7
	154.5	177.1	137.0
	154.7	177.2	137.4
M05-2X	141.4	176.0	167.6
	146.7	177.8	164.6
	147.6	178.0	163.1
	148.0	177.9	164.3

## Data Availability

Not applicable.

## Supplementary Materials

The following supporting information can be downloaded at: https://www.mdpi.com/article/10.3390/molecules28124812/s1, B3LYP/6-31 + g(d) Optimized Tetraamide Monomer.

## References

[1] Steed J.W., Atwood J.L. (2000). Supramolecular Chemistry.

[2] Bhalla V. (2018). Supramolecular Chemistry. Reson.

[3] Huang F., Anslyn E.V. (2015). Introduction: Supramolecular Chemistry. Chem. Rev..

[4] Menger F.M. (2002). Supramolecular chemistry and self-assembly. Proc. Natl. Acad. Sci. USA.

[5] Williams G.T., Haynes C.J.E., Fares M., Caltagirone C., Hiscock J.R., Gale P.A. (2021). Advances in applied supramolecular technologies. Chem. Soc. Rev..

[6] Savyasachi A.J., Kotova O., Shanmugaraju S., Bradberry S.J., Ó’máille G.M., Gunnlaugsson T. (2017). Supramolecular Chemistry: A Toolkit for Soft Functional Materials and Organic Particles. Chem.

[7] Cragg P.G. (2005). A Practical Guide to Supramolecular Chemistry.

[8] Ollerton K., Greenaway R.L., Slater A.G. (2021). Enabling Technology for Supramolecular Chemistry. Front. Chem..

[9] Lehn J.-M. (2009). Towards Complex Matter: Supramolecular Chemistry and Self-organization. Eur. Rev..

[10] Cui H., Xu B. (2017). Supramolecular medicine. Chem. Soc. Rev..

[11] Scanlon S., Aggeli A. (2008). Self-assembling peptide nanotubes. Nano Today.

[12] Mattia E., Otto S. (2015). Supramolecular systems chemistry. Nat. Nanotechnol..

[13] Guo C., Sedwick A.C., Hirao T., Sessler J.L. (2021). Supramolecular fluorescent sensors: An historical overview and update. Coord. Chem. Rev..

[14] Insua I., Montenegro J. (2020). Synthetic Supramolecular Systems in Life-like Materials and Protocell Models. Chem.

[15] Brzechwa-Chodzyńska A., Markiewicz G., Cecot P., Harrowfield J., Stefankiewicz A.R. (2023). Self-assembly of a fluorescent hydrogen-bonded capsule based on an amino-acid functionalised tetraphenylethylene. Chem. Commun..

[16] He Y., Zhang Y., Liu M., Zhao K., Shan C., Wojtas L., Guo H., Ding A., Shi X. (2021). Synthesis of microporous hydrogen-bonded supramolecular organic frameworks through guanosine self-assembly. Cell Rep. Phys. Sci..

[17] Li Z.-T., Hou J.-L., Li C., Yi H.-P. (2006). Shape-Persistent Aromatic Amide Oligomers: New Tools for Supramolecular Chemistry. Chem..

[18] Woods J.F., Gallego L., Pfister P., Maaloum M., Jentzsch A.V., Rickhaus M. (2022). Shape-assisted self-assembly. Nat. Commun..

[19] Brunsveld L., Folmer B.J.B., Meijer E.W., Sijbesma R.P. (2001). Supramolecular Polymers. Chem. Rev..

[20] Bazzicalupi C., Bianchi A., García-España E., Delgado-Pinar E. (2014). Metals in supramolecular chemistry. Inorganica Chim. Acta.

[21] Li Y., Yu H., Xu F., Guo Q., Xie Z., Sun Z. (2019). Solvent controlled self-assembly of π-stacked/H-bonded supramolecular organic frameworks from a *C*_3_-symmetric monomer for iodine adsorption. Crystengcomm.

[22] Busschaert N., Caltagirone C., Van Rossom W., Gale P.A. (2015). Applications of Supramolecular Anion Recognition. Chem. Rev..

[23] Müller-Dethlefs K., Hobza P. (2000). Noncovalent Interactions: A Challenge for Experiment and Theory. Chem. Rev..

[24] Johnson E.R., Keinan S., Mori-Sánchez P., Contreras-García J., Cohen A.J., Yang W. (2010). Revealing Noncovalent Interactions. J. Am. Chem. Soc..

[25] Jin M.Y., Zhen Q., Xiao D., Tao G., Xing X., Yu P., Xu C. (2022). Engineered non-covalent π interactions as key elements for chiral recognition. Nat. Commun..

[26] Dong W., Li Q., Scheiner S. (2018). Comparative Strengths of Tetrel, Pnicogen, Chalcogen, and Halogen Bonds and Contributing Factors. Molecules.

[27] Metrangolo P., Meyer F., Pilati T., Resnati G., Terraneo G. (2008). Halogen Bonding in Supramolecular Chemistry. Angew. Chem. Int. Ed..

[28] Parra R.D., Castillo A. (2017). Cyclic networks of halogen-bonding interactions in molecular self-assemblies: A theoretical N-X···N versus C-X···N investigation. Acta Cryst. B.

[29] Palmore G.T.S., McDonald J.C., Greenberg A., Breneman C.M., Liebman J.F. (2000). The role of amides in the noncovalent synthesis of supramolecular structures in solutions, at interfaces and in solids. The Amide Linkage: Structural Significance in Chemistry, Biochemistry, and Materials Science.

[30] Rajagopal K., Schneider J.P. (2004). Self-assembling peptides and proteins for nanotechnological applications. Curr. Opin. Struct. Biol..

[31] Liu Z., Zhou Y., Yuan L. (2022). Hydrogen-bonded aromatic amide macrocycles: Synthesis, properties and functions. Org. Biomol. Chem..

[32] Chmielewski M.J., Szumna A., Jurczak J. (2004). Anion induced conformational switch of a macrocyclic amide receptor. Tetrahedron Lett..

[33] Parra R.D., Cedeño D.L. (2007). Preferred conformations of the gas phase complex between Li+ and a model macrocycle tetraamide. J. Mol. Struct. (Theochem).

[34] Lankshear M.D., Cowley A.R., Beer P.D. (2006). Cooperative AND receptor for ion-pairs. Chem. Commun..

[35] Redman J.E., Beer P.D., Dent S.W., Drew M.G.B. (1998). Cooperative binding of potassium cation and chloride anion by novel rhenium(I) bipyridyl amide crown ether receptors. Chem. Commun..

[36] Kim Y.H., Calabrese J., McEwen C. (1996). CaCl_3_- or Ca_2_Cl_4_ complexing cyclic aromatic amide. Template effect on cyclization. J. Am. Chem. Soc..

[37] Ghorbanpour M., Wemhoff M.P., Kofoed P., Parra R.D. (2007). Intramolecular Hydrogen Bonding Effects on Chelating Binding of Ions by Aromatic Amides: A DFT Study. J. Undergrad. Chem. Res..

[38] Oschetzki D., Rauhut G. (2014). Pushing the limits in accurate vibrational structure calculations: Anharmonic frequencies of lithium fluoride clusters (LiF)n, n = 2 − 10. Phys. Chem. Chem. Phys..

[39] Doll K., Schön J.C., Jansen M. (2010). Ab initio energy landscape of LiF clusters. J. Chem. Phys..

[40] Lintuluoto M. (2001). Theoretical study on the structure and energetics of alkali halide clusters. J. Mol. Struct. (Theochem).

[41] Kollman P.A., Liebman J.F., Allen L.C. (1970). The Lithium Bond. J. Am. Chem. Soc..

[42] Baskin C.P., Bender C.F., Kollman P.A. (1973). Dimers of lithium fluoride and sodium hydride. J. Am. Chem. Soc..

[43] Kima S.K., Sessler J.L. (2010). Ion pair receptors. Chem. Soc. Rev..

[44] Tay H.M., Tse Y.C., Docker A., Gateley C., Thompson A.L., Kuhn H., Zhang Z., Beer P.D. (2023). Halogen-Bonding Heteroditopic [2]Catenanes for Recognition of Alkali Metal/Halide Ion Pairs. Angew. Chem. Int. Ed..

[45] West W.C., Whitacre J.F., Leifer N., Greenbaum S., Smart M., Bugga R., Blanco M., Narayanan S.R. (2007). Reversible Intercalation of Fluoride-Anion Receptor Complexes in Graphite. J. Electrochem. Soc..

[46] Guo X., Xu H., Li W., Liu Y., Shi Y., Li Q., Pang H. (2023). Embedding Atomically Dispersed Iron Sites in Nitrogen-Doped Carbon Frameworks-Wrapped Silicon Suboxide for Superior Lithium Storage. Adv. Sci..

[47] Xiao W., Kiran G.K., Yoo K., Kim J.-H., Xu H. (2023). The Dual-Site Adsorption and High Redox Activity Enabled by Hybrid Organic-Inorganic Vanadyl Ethylene Glycolate for High-Rate and Long-Durability Lithium–Sulfur Batteries. Small.

[48] Rao L., Ke H., Fu G., Xu X., Yan Y. (2009). Performance of Several Density Functional Theory Methods on Describing Hydrogen-Bond Interactions. J. Chem. Theory Comput..

[49] Xu X., Goddard W.A. (2004). Bonding Properties of the Water Dimer:  A Comparative Study of Density Functional Theories. J. Phys. Chem. A.

[50] Frey J.A., Leutwyler S. (2005). Binding Energies of Hydrogen-Bonded cis-Amide and Nucleobase Dimers: An Evaluation of DFT Performance. Chimia.

[51] Frisch M.J., Trucks G.W., Schlegel H.B., Scuseria G.E., Robb M.A., Cheeseman J.R., Scalmani G., Barone V., Petersson G.A., Nakatsuji H. (2016). Gaussian 16, Revision A.03.

[52] Boys S.F., Bernardi F.D. (1970). The Calculation of small molecular interactions by the differences of separate total energies. Some procedures with reduced errors. Mol. Phys..

[53] Becke A.D. (1993). Density-functional thermochemistry. III. The role of exact exchange. J. Chem. Phys..

[54] Lee C., Yang W., Parr R.G. (1988). Development of the Colle-Salvetti correlation-energy formula into a functional of the electron density. Phys. Rev. B.

[55] Vosko S.H., Wilk L., Nusair M. (1980). Accurate spin-dependent electron liquid correlation energies for local spin density calculations: A critical analysis. Can. J. Phys..

[56] Stephens P.J., Devlin F.J., Ashvar C.S., Chabalowski C.F., Frisch M.J. (1994). Theoretical calculation of vibrational circular dichroism spectra. J. Phys. Chem..

[57] Tirado-Rives J., Jorgensen W.L. (2008). Performance of B3LYP Density Functional Methods for a Large Set of Organic Molecules. J. Chem. Theory Comput..

[58] Zheng Y.-Z., Xu J., Liang Q., Da-Fu C., Rui G., Zhong-Min F. (2017). A density functional theory study on the hydrogen bonding interactions between luteolin and ethanol. J. Mol. Model..

[59] Burns L.A., Vázquez-Mayagoitia Á., Sumpter B.G., Sherrill C.D. (2011). Density-functional approaches to noncovalent interactions: A comparison of dispersion corrections (DFT-D), exchange-hole dipole moment (XDM) theory, and specialized functionals. J. Chem. Phys..

[60] Goerigk L., Hansen A., Bauer C., Ehrlich S., Najibi A., Grimme S. (2017). A look at the density functional theory zoo with the advanced GMTKN55 database for general main group thermochemistry, kinetics and noncovalent interactions. Phys. Chem. Chem. Phys..

[61] Minenkov Y., Singstad Å., Occhipinti G., Jensen V.R. (2012). The accuracy of DFT-optimized geometries of functional transition metal compounds: A validation study of catalysts for olefin metathesis and other reactions in the homogeneous phase. Dalton Trans..

[62] Tsuzuki S., Uchimaru T. (2020). Accuracy of intermolecular interaction energies, particularly those of hetero-atom containing molecules obtained by DFT calculations with Grimme’s D2, D3 and D3BJ dispersion corrections. Phys. Chem. Chem. Phys..

[63] Grimme S. (2006). Semiempirical GGA-type density functional constructed with a long-range dispersion correction. J. Comput. Chem..

[64] Grimme S., Antony J., Ehrlich S., Krieg H. (2010). A consistent and accurate ab initio parametrization of density functional dispersion correction (DFT-D) for the 94 elements H-Pu. J. Chem. Phys..

[65] Grimme S., Ehrlich S., Goerigk L. (2011). Effect of the damping function in dispersion corrected density functional theory. J. Comput. Chem..

[66] Chai J.-D., Head-Gordon M. (2008). Long-range corrected hybrid density functionals with damped atom-atom dispersion corrections. Phys. Chem. Chem. Phys..

[67] Halsey-Moorea C., Jena P., McLeskey J.T. (2019). Tuning range-separated DFT functionals for modeling the peak absorption of MEH-PPV polymer in various solvents. Comput. Theor. Chem..

[68] Kozuch S., Martin J.M.L. (2013). Halogen Bonds: Benchmarks and Theoretical Analysis. J. Chem. Theory Comput..

[69] Zhao Y., Schultz N.E., Truhlar D.G. (2006). Design of Density Functionals by Combining the Method of Constraint Satisfaction with Parametrization for Thermochemistry, Thermochemical Kinetics, and Noncovalent Interactions. J. Chem. Theory Comput..

[70] Zhao Y., Truhlar D.G. (2006). A Density Functional That Accounts for Medium-Range Correlation Energies in Organic Chemistry. Org. Lett..

[71] Zhao Y., Truhlar D.G. (2011). Applications and validations of the Minnesota density functionals. Chem. Phys. Lett..

[72] Chill S.T., Sherril C.D. (2008). Assessment of the Performance of the M05-2X and M06-2X Exchange-Correlation Functionals for Noncovalent Interactions in Biomolecules. J. Chem. Theory Comput..

[73] Josa D., Rodríguez-Otero J., Cabaleiro-Lago E.M., Rellán-Piñeiro M. (2013). Analysis of the performance of DFT-D, M05-2X and M06-2X functionals for studying π⋯π interactions. Chem. Phys. Lett..

[74] Scheiner S., Elliot R.B. (2021). 2- Insights into the nature of non-covalent bonds accessible by quantum calculations. Developments in Physical & Theoretical Chemistry, Intra-and Intermolecular Interactions between Non-Covalently Bonded Species.

[75] León I., Lesarri A., Fernández J.A., Elliot R.B. (2020). 5-Noncovalent interactions in isolated molecular aggregates: From single molecules to nanostructures. Developments in Physical & Theoretical Chemistry, Intra-and Intermolecular Interactions between Non-Covalently Bonded Species.

[76] Samantray S., Krishnaswamy S., Chand D.K. (2020). Self-assembled conjoined-cages. Nat. Commun..

[77] Vasdev R., Preston D., Crowley J.D. (2017). Multicavity Metallosupramolecular Architectures. Chem. –Asian J..

[78] Schmidt A.A.S., Casini A., Kühn F.E. (2014). Self-assembled M2L4 coordination cages: Synthesis and potential applications. Coord. Chem. Rev..

[79] Han M., Engelhard D.M., Clever G.H. (2014). Self-assembled coordination cages based on banana-shaped ligands. Chem. Soc. Rev..

