# Supplementary material for: Bracelet-like Complexes of Lithium Fluoride with Aromatic Tetraamides, and Their Potential for LiF-Mediated Self-Assembly: A DFT Study
Source: Molecules. 2023 Jun 16;28(12):4812. doi: 10.3390/molecules28124812 (PMC10303905; doi:10.3390/molecules28124812)
Supplement: Supplementary file 1 [file molecules-28-04812-s001.zip › molecules-2444250-supplementary.pdf]

B3LYP/6-31+g(d) Optimized Tetraamide Monomer

C	-0.46936200	-0.03832200	1.22020400
C	0.96012700	-0.04692900	1.23176300
C	1.61984100	-0.06317400	0.00000000
C	0.96012700	-0.04692900	-1.23176300
C	-0.46936200	-0.03832200	-1.22020400
C	-1.15900100	-0.00924500	0.00000000
H	2.69957500	-0.16928900	0.00000000
H	-2.23867100	0.01324600	0.00000000
C	1.73532500	-0.06226400	2.50519200
O	1.24720000	-0.40513300	3.59109000
N	3.06014100	0.29649700	2.44341700
H	3.52507700	0.36753800	3.34036900
C	1.73532500	-0.06226400	-2.50519200
O	1.24720000	-0.40513300	-3.59109000
N	3.06014100	0.29649700	-2.44341700
H	3.39859800	0.91154100	-1.71786600
N	-1.14550400	-0.07631600	2.43796300
C	-2.49569200	0.03197700	2.69392900
H	-0.53335000	-0.24274500	3.24009800
O	-3.38397100	0.21483400	1.88295900
H	-2.69398200	-0.06669900	3.77670500
N	-1.14550400	-0.07631600	-2.43796300
C	-2.49569200	0.03197700	-2.69392900
H	-0.53335000	-0.24274500	-3.24009800
O	-3.38397100	0.21483400	-1.88295900
H	-2.69398200	-0.06669900	-3.77670500
H	3.39859800	0.91154100	1.71786600
H	3.52507700	0.36753800	-3.34036900

B3LYPD/6-311+G(d,p)) Optimized Tetraamine Monomer

C	-0.46774700	-0.05170400	1.21818200
C	0.95843400	-0.05277500	1.22814400
C	1.61707900	-0.06807400	0.00000000
C	0.95843400	-0.05277500	-1.22814400
C	-0.46774700	-0.05170400	-1.21818200
C	-1.15476800	-0.02704600	0.00000000
H	2.69516000	-0.16705600	0.00000000
H	-2.23160500	-0.00853100	0.00000000
C	1.73155200	-0.06638200	2.50332600
O	1.25182500	-0.43745900	3.57545800
N	3.04043000	0.33840600	2.44636300
H	3.51432900	0.39586500	3.33555200
C	1.73155200	-0.06638200	-2.50332600
O	1.25182500	-0.43745900	-3.57545800
N	3.04043000	0.33840600	-2.44636300
H	3.36641300	0.94830600	-1.71564700
N	-1.14332400	-0.09470500	2.43476000
C	-2.49111900	0.03727900	2.69128600
H	-0.53618700	-0.27656300	3.23421900
O	-3.36971900	0.24648900	1.88733800
H	-2.68657300	-0.07110700	3.77324600
N	-1.14332400	-0.09470500	-2.43476000
C	-2.49111900	0.03727900	-2.69128600
H	-0.53618700	-0.27656300	-3.23421900
O	-3.36971900	0.24648900	-1.88733800
H	-2.68657300	-0.07110700	-3.77324600
H	3.36641300	0.94830600	1.71564700
H	3.51432900	0.39586500	-3.33555200

M052X/6-31+g(d) Optimized Tetraamide Monomer

C	-0.46807800	-0.05595500	1.21398600
C	0.95134000	-0.05741600	1.22270400
C	1.61508000	-0.07022100	0.00000000
C	0.95134000	-0.05741600	-1.22270400
C	-0.46807800	-0.05595500	-1.21398600
C	-1.15976800	-0.02783600	0.00000000
H	2.69319900	-0.17107800	0.00000000
H	-2.23606600	-0.00789700	0.00000000
C	1.72803600	-0.07347300	2.49314700
O	1.27112700	-0.49163200	3.55652700
N	3.01375400	0.37996300	2.43025800
H	3.48967800	0.43883100	3.31755000
C	1.72803600	-0.07347300	-2.49314700
O	1.27112700	-0.49163200	-3.55652700
N	3.01375400	0.37996300	-2.43025800
H	3.29144500	1.03624900	-1.72139600
N	-1.14001200	-0.09953200	2.42992400
C	-2.48378600	0.04125300	2.67059500
H	-0.54520800	-0.29662200	3.22944400
O	-3.34701500	0.26488400	1.85106900
H	-2.70458900	-0.07029900	3.74109400
N	-1.14001200	-0.09953200	-2.42992400
C	-2.48378600	0.04125300	-2.67059500
H	-0.54520800	-0.29662200	-3.22944400
O	-3.34701500	0.26488400	-1.85106900
H	-2.70458900	-0.07029900	-3.74109400
H	3.29144500	1.03624900	1.72139600
H	3.48967800	0.43883100	-3.31755000

wB97XD/6-311+G(d,p)) Optimized Tetraamide Monomer

C	-0.46617200	0.05586800	-1.21275900
C	0.95164900	0.05653900	-1.22203000
C	1.61198100	0.07136100	0.00000000
C	0.95164900	0.05653900	1.22203000
C	-0.46617200	0.05586800	1.21275900
C	-1.15425900	0.02993500	0.00000000
H	2.69171500	0.16729600	0.00000000
H	-2.23247000	0.00930000	0.00000000
C	1.72495600	0.07285500	-2.49694300
O	1.26211900	0.48094700	-3.55479000
N	3.01274200	-0.37644800	-2.43897100
H	3.49490300	-0.43041500	-3.32207600
C	1.72495600	0.07285500	2.49694300
O	1.26211900	0.48094700	3.55479000
N	3.01274200	-0.37644800	2.43897100
H	3.30842600	-1.00588400	1.71404900
N	-1.13754800	0.10101800	-2.42893100
C	-2.48015500	-0.04007600	-2.67979200
H	-0.53560900	0.30094200	-3.22470000
O	-3.34888100	-0.26544700	-1.87674700
H	-2.68444500	0.07606100	-3.75853300
N	-1.13754800	0.10101800	2.42893100
C	-2.48015500	-0.04007600	2.67979200
H	-0.53560900	0.30094200	3.22470000
O	-3.34888100	-0.26544700	1.87674700
H	-2.68444500	0.07606100	3.75853300
H	3.30842600	-1.00588400	-1.71404900
H	3.49490300	-0.43041500	3.32207600

B3LYP/6-31+g(d) Optimized mLiF-A Complex

C	-1.20379800	0.47822400	-1.07207700
C	-1.21323000	-0.88129800	-0.74196400
C	0.00025700	-1.56351200	-0.64786800
C	1.21360300	-0.88102900	-0.74168300
C	1.20398500	0.47851000	-1.07168600
C	0.00005200	1.14474400	-1.29832800
H	0.00029700	-2.59859200	-0.32205000
H	-0.00002800	2.20961200	-1.50516800
C	-2.47096500	-1.48602900	-0.16754400
O	-3.52488300	-1.57087200	-0.80056400
N	-2.29254200	-1.85880800	1.12509400
H	-3.10484800	-2.18383800	1.63595100
H	-1.48268400	-1.48617600	1.64751500
C	2.47142400	-1.48589100	-0.16758400
O	3.52498500	-1.57144400	-0.80100600
N	2.29363200	-1.85795800	1.12533400
H	3.10566500	-2.18348800	1.63625900
H	1.48331300	-1.48690600	1.64769300
N	-2.39046000	1.26300600	-0.91519100
C	-2.53506400	2.03322900	0.19757400
H	-3.23556800	0.95964100	-1.39017400
O	-1.66015300	2.21726900	1.04554200
H	-3.52416400	2.50749200	0.27902300
N	2.39052800	1.26340900	-0.91449000
C	2.53505300	2.03351800	0.19826000
H	3.23539300	0.96144900	-1.39075100
O	1.66031500	2.21678300	1.04662600
H	3.52388100	2.50836100	0.27954800
Li	-0.00037500	1.22433000	1.61776300
F	-0.00202800	-0.45608500	2.09291600

B3LYPD/6-311+g(d,p) Optimized mLiF-A Complex

F	-0.49894300	2.05754900	0.00000000
C	0.48422300	-1.08089400	-1.19994400
C	-0.87156100	-0.75200200	-1.20852000
C	-1.55393700	-0.65588400	0.00000000
C	-0.87156100	-0.75200200	1.20852000
C	0.48422300	-1.08089400	1.19994400
C	1.15124800	-1.30402000	0.00000000
H	-2.58303500	-0.32050500	0.00000000
H	2.21596400	-1.49808400	0.00000000
C	-1.46106500	-0.17502200	-2.47050600
O	-1.51306500	-0.79905500	-3.52422200
N	-1.84429000	1.11154700	-2.29246400
H	-2.16053700	1.61758400	-3.10696700
H	-1.49650500	1.62744000	-1.47034800
C	-1.46106500	-0.17502200	2.47050600
O	-1.51306500	-0.79905500	3.52422200
N	-1.84429000	1.11154700	2.29246400
H	-2.16053700	1.61758400	3.10696700
H	-1.49650500	1.62744000	1.47034800
N	1.26451600	-0.91103600	-2.38711800
C	2.00124100	0.22262400	-2.53069400
H	0.94959400	-1.37395900	-3.23155900
O	2.16415400	1.06723400	-1.65727300
H	2.46374200	0.31956300	-3.52294700
N	1.26451600	-0.91103600	2.38711800
C	2.00124100	0.22262400	2.53069400
H	0.94959400	-1.37395900	3.23155900
O	2.16415400	1.06723400	1.65727300
H	2.46374200	0.31956300	3.52294700
Li	1.20881700	1.66811100	0.00000000

M052X/6-31+g(d) Optimized mLiF-A Complex

C	-0.50671200	-1.04866800	1.19677600
C	0.85181100	-0.74262600	1.20507100
C	1.53640500	-0.64246000	0.00000000
C	0.85181100	-0.74262600	-1.20507100
C	-0.50671200	-1.04866800	-1.19677600
C	-1.18250800	-1.25294300	0.00000000
H	2.57047400	-0.32205900	0.00000000
H	-2.25050600	-1.42821600	0.00000000
C	1.44568600	-0.19347900	2.47215400
O	1.45727300	-0.82047800	3.52679700
N	1.88451000	1.06979700	2.29974600
H	2.21042700	1.56509300	3.11564500
H	1.55547000	1.59487500	1.47954100
C	1.44568600	-0.19347900	-2.47215400
O	1.45727300	-0.82047800	-3.52679700
N	1.88451000	1.06979700	-2.29974600
H	2.21042700	1.56509300	-3.11564500
H	1.55547000	1.59487500	-1.47954100
N	-1.27219200	-0.89372900	2.38927100
C	-1.98761800	0.24760900	2.53597600
H	-0.94589300	-1.35829800	3.22731700
O	-2.14402400	1.08564100	1.65327100
H	-2.44148100	0.36489700	3.52433300
N	-1.27219200	-0.89372900	-2.38927100
C	-1.98761800	0.24760900	-2.53597600
H	-0.94589300	-1.35829800	-3.22731700
O	-2.14402400	1.08564100	-1.65327100
H	-2.44148100	0.36489700	-3.52433300
Li	-1.12793600	1.57883100	0.00000000
F	0.55122100	2.02127500	0.00000000

wB97XD/6-311+g(d,p) Optimized mLiF-A Complex

F	0.58897700	2.05977100	0.00000000
C	-0.51313900	-1.04681600	1.19652900
C	0.84309500	-0.74182300	1.20435400
C	1.52458400	-0.65388000	0.00000000
C	0.84309500	-0.74182300	-1.20435400
C	-0.51313900	-1.04681600	-1.19652900
C	-1.18345100	-1.25399700	0.00000000
H	2.56053300	-0.33755000	0.00000000
H	-2.25041900	-1.43884700	0.00000000
C	1.45555300	-0.19344600	2.46662800
O	1.52671100	-0.83517100	3.50212700
N	1.84152000	1.08798300	2.30105800
H	2.18933100	1.58028900	3.10864700
H	1.51304900	1.60854900	1.47388500
C	1.45555300	-0.19344600	-2.46662800
O	1.52671100	-0.83517100	-3.50212700
N	1.84152000	1.08798300	-2.30105800
H	2.18933100	1.58028900	-3.10864700
H	1.51304900	1.60854900	-1.47388500
N	-1.28123600	-0.90102200	2.38811700
C	-2.00243900	0.23374700	2.55794300
H	-0.97677500	-1.39494900	3.21603200
O	-2.15669900	1.09024600	1.70300900
H	-2.46054400	0.31868600	3.55246400
N	-1.28123600	-0.90102200	-2.38811700
C	-2.00243900	0.23374700	-2.55794300
H	-0.97677500	-1.39494900	-3.21603200
O	-2.15669900	1.09024600	-1.70300900
H	-2.46054400	0.31868600	-3.55246400
Li	-1.11627600	1.58066000	0.00000000

B3LYP/6-31+g(d) Optimized mLiF-B Complex

C	-0.71984300	-0.22705700	1.22326900
C	0.69477300	-0.42167900	1.22875500
C	1.34134000	-0.57976100	0.00000000
C	0.69477300	-0.42167900	-1.22875500
C	-0.71984300	-0.22705700	-1.22326900
C	-1.40628600	-0.11076800	0.00000000
H	2.40180200	-0.79471800	0.00000000
H	-2.47367100	0.05567400	0.00000000
C	1.50247500	-0.41027500	2.49537500
O	1.03078800	-0.77965200	3.58144600
N	2.77291400	0.05357000	2.36532200
H	3.31491900	0.12212400	3.21805400
H	3.05372300	0.63310100	1.56551400
C	1.50247500	-0.41027500	-2.49537500
O	1.03078800	-0.77965200	-3.58144600
N	2.77291400	0.05357000	-2.36532200
H	3.31491900	0.12212400	-3.21805400
H	3.05372300	0.63310100	-1.56551400
F	2.89481300	1.72931900	0.00000000
N	-1.37080400	-0.16955700	2.45322000
C	-2.68174100	0.16068800	2.72721200
H	-0.77608800	-0.44228700	3.24186200
O	-3.52270000	0.51442500	1.92256400
H	-2.89183500	0.07066800	3.80769500
N	-1.37080400	-0.16955700	-2.45322000
C	-2.68174100	0.16068800	-2.72721200
H	-0.77608800	-0.44228700	-3.24186200
O	-3.52270000	0.51442500	-1.92256400
H	-2.89183500	0.07066800	-3.80769500
Li	1.23329400	1.73282000	0.00000000

B3LYPD/6-311+g(d,p) Optimized mLiF-B Complex

C	-0.72433400	-0.23877800	1.22101900
C	0.69000200	-0.41167000	1.22584100
C	1.33741700	-0.55624800	0.00000000
C	0.69000200	-0.41167000	-1.22584100
C	-0.72433400	-0.23877800	-1.22101900
C	-1.40952600	-0.13583200	0.00000000
H	2.39774200	-0.75789800	0.00000000
H	-2.47630400	0.01428900	0.00000000
C	1.49298600	-0.41027300	2.49538600
O	1.01856700	-0.78930400	3.56918700
N	2.76075000	0.05796700	2.37857900
H	3.30064200	0.10234900	3.23002200
H	3.06070700	0.61760100	1.57634300
C	1.49298600	-0.41027300	-2.49538600
O	1.01856700	-0.78930400	-3.56918700
N	2.76075000	0.05796700	-2.37857900
H	3.30064200	0.10234900	-3.23002200
H	3.06070700	0.61760100	-1.57634300
F	2.94166400	1.69731500	0.00000000
N	-1.37931100	-0.19247800	2.44817500
C	-2.68249900	0.16575100	2.72094000
H	-0.79042700	-0.47103600	3.23574100
O	-3.50740600	0.54700300	1.92310400
H	-2.89338500	0.06703300	3.80019100
N	-1.37931100	-0.19247800	-2.44817500
C	-2.68249900	0.16575100	-2.72094000
H	-0.79042700	-0.47103600	-3.23574100
O	-3.50740600	0.54700300	-1.92310400
H	-2.89338500	0.06703300	-3.80019100
Li	1.28286100	1.82932300	0.00000000

M052X/6-31+g(d) Optimized mLiF-B Complex

C	-0.68798900	-0.29673600	1.21746300
C	0.71799800	-0.47747600	1.21908200
C	1.37367500	-0.61674900	0.00000000
C	0.71799800	-0.47747600	-1.21908200
C	-0.68798900	-0.29673600	-1.21746300
C	-1.37828400	-0.17981300	0.00000000
H	2.44270300	-0.77441900	0.00000000
H	-2.44277400	-0.01610500	0.00000000
C	1.52326200	-0.40451900	2.48204100
O	1.10393200	-0.84496500	3.55385000
N	2.71903200	0.20332800	2.33868400
H	3.26844700	0.31980100	3.17640100
H	2.90092200	0.81295300	1.53726100
C	1.52326200	-0.40451900	-2.48204100
O	1.10393200	-0.84496500	-3.55385000
N	2.71903200	0.20332800	-2.33868400
H	3.26844700	0.31980100	-3.17640100
H	2.90092200	0.81295300	-1.53726100
N	-1.33211100	-0.24035700	2.44699700
C	-2.62041600	0.15774200	2.70718800
H	-0.75565700	-0.53343700	3.23332700
O	-3.41660800	0.56730500	1.89168400
H	-2.85803500	0.06981500	3.77529900
N	-1.33211100	-0.24035700	-2.44699700
C	-2.62041600	0.15774200	-2.70718800
H	-0.75565700	-0.53343700	-3.23332700
O	-3.41660800	0.56730500	-1.89168400
H	-2.85803500	0.06981500	-3.77529900
F	2.52338000	1.83668500	0.00000000
Li	0.86587100	1.63810400	0.00000000

wB97XD/6-311+g(d,p) Optimized mLiF-B Complex

C	0.69398500	0.30031600	1.21532800
C	-0.70824600	0.48604700	1.21725200
C	-1.35950500	0.63076300	0.00000000
C	-0.70824600	0.48604700	-1.21725200
C	0.69398500	0.30031600	-1.21532800
C	1.38054500	0.18443200	0.00000000
H	-2.42827100	0.79351600	0.00000000
H	2.44605700	0.01581700	0.00000000
C	-1.51189800	0.41147000	2.48256900
O	-1.09119400	0.84515500	3.54986500
N	-2.70675700	-0.20051000	2.34177900
H	-3.25787600	-0.31450800	3.17761300
H	-2.90309700	-0.79262100	1.52846400
C	-1.51189800	0.41147000	-2.48256900
O	-1.09119400	0.84515500	-3.54986500
N	-2.70675700	-0.20051000	-2.34177900
H	-3.25787600	-0.31450800	-3.17761300
H	-2.90309700	-0.79262100	-1.52846400
F	-2.61299700	-1.81806900	0.00000000
N	1.33924100	0.24467900	2.44559900
C	2.62113400	-0.16643500	2.71711000
H	0.75913400	0.54358500	3.22862200
O	3.41959600	-0.58827400	1.91998100
H	2.84394800	-0.07054200	3.79314000
N	1.33924100	0.24467900	-2.44559900
C	2.62113400	-0.16643500	-2.71711000
H	0.75913400	0.54358500	-3.22862200
O	3.41959600	-0.58827400	-1.91998100
H	2.84394800	-0.07054200	-3.79314000
Li	-0.92005900	-1.72498300	0.00000000

B3LYP/6-31+g(d) Optimized mLiF-C Complex

F	-1.08628900	2.26103900	0.00000000
C	-0.28573100	-0.62169400	1.21083700
C	1.09215200	-0.30993400	1.22701700
C	1.75335600	-0.19369400	0.00000000
C	1.09215200	-0.30993400	-1.22701700
C	-0.28573100	-0.62169400	-1.21083700
C	-0.93586700	-0.84355700	0.00000000
H	2.82648900	-0.02787800	0.00000000
H	-1.97316100	-1.14394500	0.00000000
C	1.80060400	-0.09862700	2.52417700
O	1.43164100	-0.64262800	3.57194100
N	2.90471900	0.71042000	2.49804300
H	3.30740500	0.92606000	3.40242900
H	2.98137700	1.43206400	1.79385600
C	1.80060400	-0.09862700	-2.52417700
O	1.43164100	-0.64262800	-3.57194100
N	2.90471900	0.71042000	-2.49804300
H	3.30740500	0.92606000	-3.40242900
H	2.98137700	1.43206400	-1.79385600
N	-1.02860000	-0.67505900	2.40624400
C	-2.28805000	-0.17450700	2.55905800
H	-0.47570900	-0.85598100	3.24526300
O	-2.99752200	0.30119200	1.67454200
H	-2.63916000	-0.24134400	3.60001700
N	-1.02860000	-0.67505900	-2.40624400
C	-2.28805000	-0.17450700	-2.55905800
H	-0.47570900	-0.85598100	-3.24526300
O	-2.99752200	0.30119200	-1.67454200
H	-2.63916000	-0.24134400	-3.60001700
Li	-2.45625000	1.31648600	0.00000000

B3LYP/6-31+g(d) Optimized $(m\text{-}LiF)_2$-A Complex

C	-0.83184000	3.81994800	-1.20226500
C	0.56737300	3.85167900	-1.21267900
C	1.25139600	3.92300800	0.00090100
C	0.56840100	3.84809400	1.21483800
C	-0.83085100	3.81637800	1.20549900
C	-1.53222400	3.85546800	0.00196700
H	2.33587600	3.88431600	0.00037700
H	-2.61457600	3.78881000	0.00232400
C	1.29837000	3.50242500	-2.48217300
O	1.15912700	4.13335500	-3.53222500
N	2.07306500	2.39635000	-2.33191700
H	2.51710100	2.01826700	-3.15992000
H	1.88499000	1.76375900	-1.54579000
C	1.30044000	3.49570000	2.48279600
O	1.16090400	4.12316600	3.53489100
N	2.07676600	2.39122400	2.32896600
H	2.52151000	2.01131900	3.15574800
H	1.88934500	1.76038600	1.54145700
N	-1.55222800	3.52461400	-2.39990600
C	-2.07842300	2.28035900	-2.58159200
H	-1.32083200	4.05895000	-3.23268600
O	-2.10213300	1.38940700	-1.73411800
H	-2.50883900	2.13675700	-3.58515300
N	-1.55028900	3.51769200	2.40286500
C	-2.07676600	2.27308500	2.58132900
H	-1.31776700	4.04936800	3.23703800
O	-2.10155600	1.38459800	1.73129800
H	-2.50613600	2.12666900	3.58494100
Li	-0.98915700	0.79169200	-0.00341500
F	0.90765200	1.00843200	-0.00429400

C	0.83184000	-3.81994800	-1.20226500
C	-0.56737300	-3.85167900	-1.21267900
C	-1.25139600	-3.92300800	0.00090100
C	-0.56840100	-3.84809400	1.21483800
C	0.83085100	-3.81637800	1.20549900
C	1.53222400	-3.85546800	0.00196700
H	-2.33587600	-3.88431600	0.00037700
H	2.61457600	-3.78881000	0.00232400
C	-1.29837000	-3.50242500	-2.48217300
O	-1.15912700	-4.13335500	-3.53222500
N	-2.07306500	-2.39635000	-2.33191700
H	-2.51710100	-2.01826700	-3.15992000
H	-1.88499000	-1.76375900	-1.54579000
C	-1.30044000	-3.49570000	2.48279600
O	-1.16090400	-4.12316600	3.53489100
N	-2.07676600	-2.39122400	2.32896600
H	-2.52151000	-2.01131900	3.15574800
H	-1.88934500	-1.76038600	1.54145700
N	1.55222800	-3.52461400	-2.39990600
C	2.07842300	-2.28035900	-2.58159200
H	1.32083200	-4.05895000	-3.23268600
O	2.10213300	-1.38940700	-1.73411800
H	2.50883900	-2.13675700	-3.58515300
N	1.55028900	-3.51769200	2.40286500
C	2.07676600	-2.27308500	2.58132900
H	1.31776700	-4.04936800	3.23703800
O	2.10155600	-1.38459800	1.73129800
H	2.50613600	-2.12666900	3.58494100
Li	0.98915700	-0.79169200	-0.00341500
F	-0.90765200	-1.00843200	-0.00429400

B3LYPD/6-311+g(d,p) Optimized (m-LiF)$_2$-A Complex

C	-0.85987200	3.87858300	-1.06532800
C	0.53420700	3.89818600	-1.12007400
C	1.25993000	3.82652400	0.06444000
C	0.61748700	3.62272600	1.28106300
C	-0.77765800	3.60713700	1.31836400
C	-1.51833200	3.78414600	0.15543600
H	2.33964900	3.76491600	0.01994800
H	-2.59780000	3.71707800	0.18485400
C	1.20505900	3.67399300	-2.44951600
O	1.03556700	4.41356900	-3.41241900
N	1.94747400	2.53980200	-2.45140000
H	2.35089600	2.24845300	-3.32960300
H	1.78118700	1.84470600	-1.71656300
C	1.37474500	3.11177000	2.47769900
O	1.26225500	3.60548500	3.59492700
N	2.12299100	2.02384500	2.17483000
H	2.57563700	1.53988100	2.93548100
H	1.91539800	1.50103000	1.32165100
F	0.88851200	1.01912900	-0.27452700
N	-1.61885700	3.70716800	-2.26338000
C	-2.12299100	2.47817200	-2.56088900
H	-1.41582100	4.32732600	-3.03860700
O	-2.09740000	1.50845300	-1.81487400
H	-2.58031500	2.43145500	-3.56036300
N	-1.45454300	3.17316000	2.49862300
C	-1.96585100	1.91348500	2.55079500
H	-1.18062700	3.60022400	3.37595200
O	-2.01982100	1.13387000	1.60857600
H	-2.34909500	1.64978600	3.54831600
Li	-0.98687500	0.77401800	-0.23421800

C	0.85987200	-3.87858300	-1.06532800
C	-0.53420700	-3.89818600	-1.12007400
C	-1.25993000	-3.82652400	0.06444000
C	-0.61748700	-3.62272600	1.28106300
C	0.77765800	-3.60713700	1.31836400
C	1.51833200	-3.78414600	0.15543600
H	-2.33964900	-3.76491600	0.01994800
H	2.59780000	-3.71707800	0.18485400
C	-1.20505900	-3.67399300	-2.44951600
O	-1.03556700	-4.41356900	-3.41241900
N	-1.94747400	-2.53980200	-2.45140000
H	-2.35089600	-2.24845300	-3.32960300
H	-1.78118700	-1.84470600	-1.71656300
C	-1.37474500	-3.11177000	2.47769900
O	-1.26225500	-3.60548500	3.59492700
N	-2.12299100	-2.02384500	2.17483000
H	-2.57563700	-1.53988100	2.93548100
H	-1.91539800	-1.50103000	1.32165100
F	-0.88851200	-1.01912900	-0.27452700
N	1.61885700	-3.70716800	-2.26338000
C	2.12299100	-2.47817200	-2.56088900
H	1.41582100	-4.32732600	-3.03860700
O	2.09740000	-1.50845300	-1.81487400
H	2.58031500	-2.43145500	-3.56036300
N	1.45454300	-3.17316000	2.49862300
C	1.96585100	-1.91348500	2.55079500
H	1.18062700	-3.60022400	3.37595200
O	2.01982100	-1.13387000	1.60857600
H	2.34909500	-1.64978600	3.54831600
Li	0.98687500	-0.77401800	-0.23421800

M052X/6-31+g(d) Optimized (m-LiF)$_2$-A Complex

C	-3.68105000	-1.16859800	0.84537400
C	-3.71249100	-1.19225300	-0.54703800
C	-3.74615700	0.00639500	-1.24855200
C	-3.65829800	1.21778500	-0.57444700
C	-3.62699200	1.22415300	0.81817700
C	-3.68185500	0.03645500	1.53611400
H	-3.69077800	-0.00705100	-2.32971800
H	-3.60735500	0.04705700	2.61548400
C	-3.38260200	-2.48120000	-1.24279200
O	-4.01594800	-3.51680600	-1.05941600
N	-2.28931500	-2.36161400	-2.02501100
H	-1.92534400	-3.20033200	-2.44992700
H	-1.65863600	-1.57346700	-1.86179500
C	-3.27172700	2.47537800	-1.29803800
O	-3.86014700	3.54125600	-1.13896800
N	-2.18297200	2.29165800	-2.07346300
H	-1.77972600	3.10403300	-2.51365900
H	-1.58569800	1.48221300	-1.89190900
N	-3.43048600	-2.36941200	1.56712300
C	-2.18980000	-2.57267700	2.07539200
H	-3.97335600	-3.18587300	1.31424200
O	-1.29573900	-1.73473200	2.09324800
H	-2.04832100	-3.57434500	2.49459600
N	-3.32353400	2.42916700	1.51199700
C	-2.07570800	2.59010200	2.01782800
H	-3.82928400	3.26259000	1.23805100
O	-1.21889300	1.71483800	2.05891700
H	-1.89162700	3.59527700	2.41171800
Li	-0.79814700	-0.04359900	0.96497000
F	-0.98955500	-0.05617500	-0.89657400

C	3.67988800	-1.17085300	-0.84529700
C	3.71117900	-1.19442400	0.54714400
C	3.74611700	0.00422700	1.24854200
C	3.65960600	1.21566700	0.57431500
C	3.62828400	1.22194300	-0.81827600
C	3.68193100	0.03413700	-1.53613300
H	3.69066100	-0.00901400	2.32970600
H	3.60747100	0.04470500	-2.61550600
C	3.37996000	-2.48299900	1.24297400
O	4.01221900	-3.51926200	1.05963900
N	2.28669300	-2.36233200	2.02507200
H	1.92175700	-3.20064600	2.44995200
H	1.65689200	-1.57349700	1.86203100
C	3.27435200	2.47368200	1.29786300
O	3.86401300	3.53889700	1.13894900
N	2.18534100	2.29107900	2.07321400
H	1.78328800	3.10386500	2.51373200
H	1.58723100	1.48230000	1.89132300
N	3.42827200	-2.37148600	-1.56696700
C	2.18744700	-2.57375300	-2.07529500
H	3.97053800	-3.18838200	-1.31422900
O	1.29405400	-1.73512200	-2.09319000
H	2.04518400	-3.57536300	-2.49438900
N	3.32593400	2.42725700	-1.51212500
C	2.07811600	2.58951400	-2.01746600
H	3.83262700	3.26015000	-1.23828700
O	1.22041400	1.71507100	-2.05837900
H	1.89489700	3.59484000	-2.41134500
Li	0.79792900	-0.04364200	-0.96564900
F	0.98952400	-0.05457600	0.89594600

wB97XD/6-311+g(d,p) Optimized (m-LiF)$_2$-A Complex

C	-0.83098900	3.75704100	-1.13106800
C	0.55911700	3.76830000	-1.16355700
C	1.26442100	3.75734200	0.02991200
C	0.60296100	3.62832300	1.24127700
C	-0.78758300	3.61867400	1.25803200
C	-1.50848900	3.72729000	0.07850500
H	2.34527200	3.68975600	0.00639300
H	-2.58983900	3.67727500	0.09502800
C	1.25589800	3.48220300	-2.46612800
O	1.12729400	4.18488700	-3.45615500
N	1.97371300	2.34095800	-2.40202900
H	2.40602900	2.00754200	-3.24866000
H	1.80366800	1.69229200	-1.62734400
C	1.34498000	3.19374800	2.47614100
O	1.24981100	3.77464700	3.54616200
N	2.05946400	2.07127400	2.25379400
H	2.51570900	1.63621600	3.03919700
H	1.86150000	1.51343700	1.41859300
N	-1.56816300	3.57041100	-2.33444600
C	-2.08715400	2.34728000	-2.60908000
H	-1.34141500	4.16786300	-3.11833300
O	-2.09721100	1.40544100	-1.83678400
H	-2.52136500	2.27873100	-3.61674100
N	-1.48032900	3.29759300	2.45907900
C	-2.00577500	2.05627100	2.61095800
H	-1.21377200	3.79630700	3.29770700
O	-2.06246300	1.21042600	1.73612700
H	-2.39874200	1.87670300	3.62219800
Li	-0.94841900	0.84362200	-0.11518100
F	0.94841900	0.97242300	-0.13578200

C	0.83098900	-3.75704100	-1.13106800
C	-0.55911700	-3.76830000	-1.16355700
C	-1.26442100	-3.75734200	0.02991200
C	-0.60296100	-3.62832300	1.24127700
C	0.78758300	-3.61867400	1.25803200
C	1.50848900	-3.72729000	0.07850500
H	-2.34527200	-3.68975600	0.00639300
H	2.58983900	-3.67727500	0.09502800
C	-1.25589800	-3.48220300	-2.46612800
O	-1.12729400	-4.18488700	-3.45615500
N	-1.97371300	-2.34095800	-2.40202900
H	-2.40602900	-2.00754200	-3.24866000
H	-1.80366800	-1.69229200	-1.62734400
C	-1.34498000	-3.19374800	2.47614100
O	-1.24981100	-3.77464700	3.54616200
N	-2.05946400	-2.07127400	2.25379400
H	-2.51570900	-1.63621600	3.03919700
H	-1.86150000	-1.51343700	1.41859300
N	1.56816300	-3.57041100	-2.33444600
C	2.08715400	-2.34728000	-2.60908000
H	1.34141500	-4.16786300	-3.11833300
O	2.09721100	-1.40544100	-1.83678400
H	2.52136500	-2.27873100	-3.61674100
N	1.48032900	-3.29759300	2.45907900
C	2.00577500	-2.05627100	2.61095800
H	1.21377200	-3.79630700	3.29770700
O	2.06246300	-1.21042600	1.73612700
H	2.39874200	-1.87670300	3.62219800
Li	0.94841900	-0.84362200	-0.11518100
F	-0.94841900	-0.97242300	-0.13578200

B3LYP/6-31+g(d) Optimized (m-LiF)$_2$-B Complex

C	-1.05310700	2.60406900	1.21506200
C	-2.06420000	1.61623700	1.22891100
C	-2.57790400	1.19948900	0.00000000
C	-2.06420000	1.61623700	-1.22891100
C	-1.05310700	2.60406900	-1.21506200
C	-0.61151600	3.13025800	0.00000000
H	-3.38778000	0.48599700	0.00000000
H	0.07696900	3.95721800	0.00000000
C	-2.55982600	1.00762800	2.50734500
O	-2.37955300	1.57546300	3.60309700
N	-3.19645200	-0.17833200	2.40584900
H	-3.54619700	-0.57549100	3.26842300
H	-3.24755700	-0.74679500	1.54518600
C	-2.55982600	1.00762800	-2.50734500
O	-2.37955300	1.57546300	-3.60309700
N	-3.19645200	-0.17833200	-2.40584900
H	-3.54619700	-0.57549100	-3.26842300
H	-3.24755700	-0.74679500	-1.54518600
F	-3.24578300	-1.64120200	0.00000000
N	-0.49111600	3.05899500	2.42313600
C	0.75909800	3.54965400	2.59603200
H	-1.04350200	2.79154900	3.24883500
O	1.60825000	3.74221400	1.71830300
H	0.97739700	3.77950300	3.64911300
N	-0.49111600	3.05899500	-2.42313600
C	0.75909800	3.54965400	-2.59603200
H	-1.04350200	2.79154900	-3.24883500
O	1.60825000	3.74221400	-1.71830300
H	0.97739700	3.77950300	-3.64911300
Li	2.30087600	3.07762700	0.00000000

C	1.05310700	-2.60406900	1.21506200
C	2.06420000	-1.61623700	1.22891100
C	2.57790400	-1.19948900	0.00000000
C	2.06420000	-1.61623700	-1.22891100
C	1.05310700	-2.60406900	-1.21506200
C	0.61151600	-3.13025800	0.00000000
H	3.38778000	-0.48599700	0.00000000
H	-0.07696900	-3.95721800	0.00000000
C	2.55982600	-1.00762800	2.50734500
O	2.37955300	-1.57546300	3.60309700
N	3.19645200	0.17833200	2.40584900
H	3.54619700	0.57549100	3.26842300
H	3.24755700	0.74679500	1.54518600
C	2.55982600	-1.00762800	-2.50734500
O	2.37955300	-1.57546300	-3.60309700
N	3.19645200	0.17833200	-2.40584900
H	3.54619700	0.57549100	-3.26842300
H	3.24755700	0.74679500	-1.54518600
F	3.24578300	1.64120200	0.00000000
N	0.49111600	-3.05899500	2.42313600
C	-0.75909800	-3.54965400	2.59603200
H	1.04350200	-2.79154900	3.24883500
O	-1.60825000	-3.74221400	1.71830300
H	-0.97739700	-3.77950300	3.64911300
N	0.49111600	-3.05899500	-2.42313600
C	-0.75909800	-3.54965400	-2.59603200
H	1.04350200	-2.79154900	-3.24883500
O	-1.60825000	-3.74221400	-1.71830300
H	-0.97739700	-3.77950300	-3.64911300
Li	-2.30087600	-3.07762700	0.00000000

B3LYPD/6-311+g(d,p) Optimized (m-LiF)$_2$-B Complex

C	-1.53932200	1.63656700	1.21650500
C	-1.77263800	0.24192800	1.22978600
C	-1.90776700	-0.39536500	0.00000000
C	-1.77263800	0.24192800	-1.22978600
C	-1.53932200	1.63656700	-1.21650500
C	-1.49495800	2.31274200	0.00000000
H	-2.11582000	-1.45333300	0.00000000
H	-1.44720300	3.38292700	0.00000000
C	-1.92516200	-0.52655000	2.50996800
O	-1.91129000	0.05215200	3.60762000
N	-2.08038700	-1.86581400	2.42945400
H	-2.26538000	-2.33177100	3.30456000
H	-2.15479400	-2.41519000	1.56584000
C	-1.92516200	-0.52655000	-2.50996800
O	-1.91129000	0.05215200	-3.60762000
N	-2.08038700	-1.86581400	-2.42945400
H	-2.26538000	-2.33177100	-3.30456000
H	-2.15479400	-2.41519000	-1.56584000
F	-2.12388400	-3.31641200	0.00000000
N	-1.36760600	2.33891400	2.41953100
C	-0.73546800	3.52065500	2.59581800
H	-1.57336200	1.75868000	3.24468300
O	-0.26069800	4.24932100	1.72512000
H	-0.67534900	3.79908100	3.65702300
N	-1.36760600	2.33891400	-2.41953100
C	-0.73546800	3.52065500	-2.59581800
H	-1.57336200	1.75868000	-3.24468300
O	-0.26069800	4.24932100	-1.72512000
H	-0.67534900	3.79908100	-3.65702300
Li	0.66120600	4.22575600	0.00000000

C	1.53932200	-1.63656700	1.21650500
C	1.77263800	-0.24192800	1.22978600
C	1.90776700	0.39536500	0.00000000
C	1.77263800	-0.24192800	-1.22978600
C	1.53932200	-1.63656700	-1.21650500
C	1.49495800	-2.31274200	0.00000000
H	2.11582000	1.45333300	0.00000000
H	1.44720300	-3.38292700	0.00000000
C	1.92516200	0.52655000	2.50996800
O	1.91129000	-0.05215200	3.60762000
N	2.08038700	1.86581400	2.42945400
H	2.26538000	2.33177100	3.30456000
H	2.15479400	2.41519000	1.56584000
C	1.92516200	0.52655000	-2.50996800
O	1.91129000	-0.05215200	-3.60762000
N	2.08038700	1.86581400	-2.42945400
H	2.26538000	2.33177100	-3.30456000
H	2.15479400	2.41519000	-1.56584000
F	2.12388400	3.31641200	0.00000000
N	1.36760600	-2.33891400	2.41953100
C	0.73546800	-3.52065500	2.59581800
H	1.57336200	-1.75868000	3.24468300
O	0.26069800	-4.24932100	1.72512000
H	0.67534900	-3.79908100	3.65702300
N	1.36760600	-2.33891400	-2.41953100
C	0.73546800	-3.52065500	-2.59581800
H	1.57336200	-1.75868000	-3.24468300
O	0.26069800	-4.24932100	-1.72512000
H	0.67534900	-3.79908100	-3.65702300
Li	-0.66120600	-4.22575600	0.00000000

M052X/6-31+g(d) Optimized (m-LiF)$_2$-B Complex

C	-1.39042600	1.94519700	1.21352800
C	-1.71647100	0.57498800	1.22585100
C	-1.89467200	-0.05580700	0.00000000
C	-1.71647100	0.57498800	-1.22585100
C	-1.39042600	1.94519700	-1.21352800
C	-1.29339600	2.62138400	0.00000000
H	-2.17870800	-1.09566900	0.00000000
H	-1.19072000	3.69015300	0.00000000
C	-1.92814500	-0.18268100	2.50223000
O	-1.93055200	0.39827800	3.59746900
N	-2.12268400	-1.51099600	2.41763300
H	-2.36449400	-1.96148800	3.28649800
H	-2.21448400	-2.05295500	1.55338200
C	-1.92814500	-0.18268100	-2.50223000
O	-1.93055200	0.39827800	-3.59746900
N	-2.12268400	-1.51099600	-2.41763300
H	-2.36449400	-1.96148800	-3.28649800
H	-2.21448400	-2.05295500	-1.55338200
F	-2.29113800	-2.95837100	0.00000000
N	-1.19258500	2.63950700	2.41515900
C	-0.43551100	3.73983000	2.58571700
H	-1.47520200	2.10375100	3.24066700
O	0.14429500	4.38165100	1.70895500
H	-0.36926100	4.04491000	3.63431600
N	-1.19258500	2.63950700	-2.41515900
C	-0.43551100	3.73983000	-2.58571700
H	-1.47520200	2.10375100	-3.24066700
O	0.14429500	4.38165100	-1.70895500
H	-0.36926100	4.04491000	-3.63431600
Li	1.01829400	4.09477900	0.00000000

C	1.39042600	-1.94519700	1.21352800
C	1.71647100	-0.57498800	1.22585100
C	1.89467200	0.05580700	0.00000000
C	1.71647100	-0.57498800	-1.22585100
C	1.39042600	-1.94519700	-1.21352800
C	1.29339600	-2.62138400	0.00000000
H	2.17870800	1.09566900	0.00000000
H	1.19072000	-3.69015300	0.00000000
C	1.92814500	0.18268100	2.50223000
O	1.93055200	-0.39827800	3.59746900
N	2.12268400	1.51099600	2.41763300
H	2.36449400	1.96148800	3.28649800
H	2.21448400	2.05295500	1.55338200
C	1.92814500	0.18268100	-2.50223000
O	1.93055200	-0.39827800	-3.59746900
N	2.12268400	1.51099600	-2.41763300
H	2.36449400	1.96148800	-3.28649800
H	2.21448400	2.05295500	-1.55338200
F	2.29113800	2.95837100	0.00000000
N	1.19258500	-2.63950700	2.41515900
C	0.43551100	-3.73983000	2.58571700
H	1.47520200	-2.10375100	3.24066700
O	-0.14429500	-4.38165100	1.70895500
H	0.36926100	-4.04491000	3.63431600
N	1.19258500	-2.63950700	-2.41515900
C	0.43551100	-3.73983000	-2.58571700
H	1.47520200	-2.10375100	-3.24066700
O	-0.14429500	-4.38165100	-1.70895500
H	0.36926100	-4.04491000	-3.63431600
Li	-1.01829400	-4.09477900	0.00000000

wB97XD/6-311+g(d,p) Optimized (m-LiF)$_2$-B Complex

C	-1.42295200	1.76124400	1.21251400
C	-1.68353300	0.37864500	1.22425400
C	-1.82455300	-0.25695800	0.00000000
C	-1.68353300	0.37864500	-1.22425400
C	-1.42295200	1.76124400	-1.21251400
C	-1.35149500	2.43616600	0.00000000
H	-2.05069500	-1.31306900	0.00000000
H	-1.29486100	3.50785700	0.00000000
C	-1.87150500	-0.38635600	2.50121400
O	-1.86412900	0.18705800	3.59409600
N	-2.05753300	-1.71659500	2.41430900
H	-2.27211900	-2.17457100	3.28520800
H	-2.15437400	-2.25821200	1.54826900
C	-1.87150500	-0.38635600	-2.50121400
O	-1.86412900	0.18705800	-3.59409600
N	-2.05753300	-1.71659500	-2.41430900
H	-2.27211900	-2.17457100	-3.28520800
H	-2.15437400	-2.25821200	-1.54826900
F	-2.20064600	-3.14892900	0.00000000
N	-1.26926200	2.45546100	2.41881100
C	-0.61688900	3.61766200	2.61021400
H	-1.50035700	1.87706300	3.23566500
O	-0.10877800	4.33215000	1.75525600
H	-0.57474900	3.89598100	3.67202800
N	-1.26926200	2.45546100	-2.41881100
C	-0.61688900	3.61766200	-2.61021400
H	-1.50035700	1.87706300	-3.23566500
O	-0.10877800	4.33215000	-1.75525600
H	-0.57474900	3.89598100	-3.67202800
Li	0.80207800	4.17866700	0.00000000

C	1.42295200	-1.76124400	1.21251400
C	1.68353300	-0.37864500	1.22425400
C	1.82455300	0.25695800	0.00000000
C	1.68353300	-0.37864500	-1.22425400
C	1.42295200	-1.76124400	-1.21251400
C	1.35149500	-2.43616600	0.00000000
H	2.05069500	1.31306900	0.00000000
H	1.29486100	-3.50785700	0.00000000
C	1.87150500	0.38635600	2.50121400
O	1.86412900	-0.18705800	3.59409600
N	2.05753300	1.71659500	2.41430900
H	2.27211900	2.17457100	3.28520800
H	2.15437400	2.25821200	1.54826900
C	1.87150500	0.38635600	-2.50121400
O	1.86412900	-0.18705800	-3.59409600
N	2.05753300	1.71659500	-2.41430900
H	2.27211900	2.17457100	-3.28520800
H	2.15437400	2.25821200	-1.54826900
F	2.20064600	3.14892900	0.00000000
N	1.26926200	-2.45546100	2.41881100
C	0.61688900	-3.61766200	2.61021400
H	1.50035700	-1.87706300	3.23566500
O	0.10877800	-4.33215000	1.75525600
H	0.57474900	-3.89598100	3.67202800
N	1.26926200	-2.45546100	-2.41881100
C	0.61688900	-3.61766200	-2.61021400
H	1.50035700	-1.87706300	-3.23566500
O	0.10877800	-4.33215000	-1.75525600
H	0.57474900	-3.89598100	-3.67202800
Li	-0.80207800	-4.17866700	0.00000000

B3LYP/6-31+G(d) Optimized [(m-LiF)$_2$]$_2$-B Complex

C	2.61035000	4.11884400	1.21545400
C	2.56617600	5.53356900	1.22929400
C	2.60075200	6.19252100	0.00000000
C	2.56617600	5.53356900	-1.22929400
C	2.61035000	4.11884400	-1.21545400
C	2.68511700	3.43680000	0.00000000
H	2.62301400	7.27136900	0.00000000
H	2.80074900	2.36896000	0.00000000
C	2.45805600	6.31497100	2.50462900
O	2.74436400	5.80100800	3.60502400
N	2.02788100	7.59110300	2.40118900
H	1.97840000	8.11807500	3.26364800
H	1.64807000	8.01786500	1.54143200
C	2.45805600	6.31497100	-2.50462900
O	2.74436400	5.80100800	-3.60502400
N	2.02788100	7.59110300	-2.40118900
H	1.97840000	8.11807500	-3.26364800
H	1.64807000	8.01786500	-1.54143200
F	0.97780700	8.62434200	0.00000000
N	2.58564400	3.40813400	2.42817900
C	2.20982600	2.12179800	2.62612100
H	2.73636600	4.01323000	3.24687400
O	1.86579800	1.30002700	1.77173300
H	2.23798600	1.84805100	3.69235900
N	2.58564400	3.40813400	-2.42817900
C	2.20982600	2.12179800	-2.62612100
H	2.73636600	4.01323000	-3.24687400
O	1.86579800	1.30002700	-1.77173300
H	2.23798600	1.84805100	-3.69235900
Li	1.00102800	0.73779600	0.00000000

C	-2.41873300	5.75764300	1.21615600
C	-2.29041300	4.34852300	1.23088100
C	-2.27841400	3.68954900	0.00000000
C	-2.29041300	4.34852300	-1.23088100
C	-2.41873300	5.75764300	-1.21615600
C	-2.54393200	6.43017900	0.00000000
H	-2.22912500	2.61105800	0.00000000
H	-2.75117000	7.48530200	0.00000000
C	-2.17503800	3.57842800	2.51341300
O	-2.47763200	4.09884600	3.60586300
N	-1.73205200	2.30614900	2.43218700
H	-1.70039600	1.78269900	3.29685900
H	-1.38584900	1.84717300	1.58167500
C	-2.17503800	3.57842800	-2.51341300
O	-2.47763200	4.09884600	-3.60586300
N	-1.73205200	2.30614900	-2.43218700
H	-1.70039600	1.78269900	-3.29685900
H	-1.38584900	1.84717300	-1.58167500
F	-0.80880300	1.08849600	0.00000000
N	-2.42542100	6.48411000	2.42086400
C	-2.05040300	7.77483500	2.59125500
H	-2.55621500	5.88626700	3.24822700
O	-1.69084700	8.56607900	1.71250300
H	-2.09293300	8.08669100	3.64497100
N	-2.42542100	6.48411000	-2.42086400
C	-2.05040300	7.77483500	-2.59125500
H	-2.55621500	5.88626700	-3.24822700
O	-1.69084700	8.56607900	-1.71250300
H	-2.09293300	8.08669100	-3.64497100
Li	-0.73475200	8.74438700	0.00000000
C	2.41873300	-5.75764300	1.21615600
C	2.29041300	-4.34852300	1.23088100

C	2.27841400	-3.68954900	0.00000000
C	2.29041300	-4.34852300	-1.23088100
C	2.41873300	-5.75764300	-1.21615600
C	2.54393200	-6.43017900	0.00000000
H	2.22912500	-2.61105800	0.00000000
H	2.75117000	-7.48530200	0.00000000
C	2.17503800	-3.57842800	2.51341300
O	2.47763200	-4.09884600	3.60586300
N	1.73205200	-2.30614900	2.43218700
H	1.70039600	-1.78269900	3.29685900
H	1.38584900	-1.84717300	1.58167500
C	2.17503800	-3.57842800	-2.51341300
O	2.47763200	-4.09884600	-3.60586300
N	1.73205200	-2.30614900	-2.43218700
H	1.70039600	-1.78269900	-3.29685900
H	1.38584900	-1.84717300	-1.58167500
F	0.80880300	-1.08849600	0.00000000
N	2.42542100	-6.48411000	2.42086400
C	2.05040300	-7.77483500	2.59125500
H	2.55621500	-5.88626700	3.24822700
O	1.69084700	-8.56607900	1.71250300
H	2.09293300	-8.08669100	3.64497100
N	2.42542100	-6.48411000	-2.42086400
C	2.05040300	-7.77483500	-2.59125500
H	2.55621500	-5.88626700	-3.24822700
O	1.69084700	-8.56607900	-1.71250300
H	2.09293300	-8.08669100	-3.64497100
Li	0.73475200	-8.74438700	0.00000000
C	-2.61035000	-4.11884400	1.21545400
C	-2.56617600	-5.53356900	1.22929400
C	-2.60075300	-6.19252100	0.00000000
C	-2.56617600	-5.53356900	-1.22929400

C	-2.61035000	-4.11884400	-1.21545400
C	-2.68511700	-3.43680000	0.00000000
H	-2.62301400	-7.27136900	0.00000000
H	-2.80074900	-2.36896000	0.00000000
C	-2.45805600	-6.31497100	2.50462900
O	-2.74436400	-5.80100800	3.60502400
N	-2.02788100	-7.59110300	2.40118900
H	-1.97840000	-8.11807500	3.26364800
H	-1.64807000	-8.01786500	1.54143200
C	-2.45805600	-6.31497100	-2.50462900
O	-2.74436400	-5.80100800	-3.60502400
N	-2.02788100	-7.59110300	-2.40118900
H	-1.97840000	-8.11807500	-3.26364800
H	-1.64807000	-8.01786500	-1.54143200
F	-0.97780700	-8.62434200	0.00000000
N	-2.58564400	-3.40813400	2.42817900
C	-2.20982600	-2.12179800	2.62612100
H	-2.73636600	-4.01323000	3.24687400
O	-1.86579800	-1.30002700	1.77173300
H	-2.23798600	-1.84805100	3.69235900
N	-2.58564400	-3.40813400	-2.42817900
C	-2.20982600	-2.12179800	-2.62612100
H	-2.73636600	-4.01323000	-3.24687400
O	-1.86579800	-1.30002700	-1.77173300
H	-2.23798600	-1.84805100	-3.69235900
Li	-1.00102800	-0.73779600	0.00000000

B3LYPD/6-311+G(d,p) Optimized [(m-LiF)$_2$]$_2$-B Complex

C	2.16525200	3.95158700	1.21671100
C	1.94504300	5.34972100	1.22950600
C	1.86526900	5.99574500	0.00000000
C	1.94504300	5.34972100	-1.22950600
C	2.16525200	3.95158700	-1.21671100
C	2.32247300	3.29218900	0.00000000
H	1.72250700	7.06418300	0.00000000
H	2.59463000	2.25691900	0.00000000
C	1.84795200	6.13076600	2.50693000
O	2.00882200	5.58133900	3.60854700
N	1.57965400	7.45226500	2.42379600
H	1.60904800	7.95286900	3.29876100
H	1.48350200	7.99795900	1.56019100
C	1.84795200	6.13076600	-2.50693000
O	2.00882200	5.58133900	-3.60854700
N	1.57965400	7.45226500	-2.42379600
H	1.60904800	7.95286900	-3.29876100
H	1.48350200	7.99795900	-1.56019100
F	1.16125700	8.84607600	0.00000000
N	2.24302300	3.24446100	2.42475300
C	2.07170400	1.91783900	2.62573100
H	2.24240500	3.86988500	3.24258100
O	1.88808800	1.04771800	1.77845900
H	2.11311400	1.66443800	3.69539500
N	2.24302300	3.24446100	-2.42475300
C	2.07170400	1.91783900	-2.62573100
H	2.24240500	3.86988500	-3.24258100
O	1.88808800	1.04771800	-1.77845900
H	2.11311400	1.66443800	-3.69539500
Li	1.09665300	0.55145800	0.00000000

C	-1.77182200	6.05787800	1.21630900
C	-1.53757200	4.66339700	1.23023100
C	-1.45711900	4.01670200	0.00000000
C	-1.53757200	4.66339700	-1.23023100
C	-1.77182200	6.05787800	-1.21630900
C	-1.94903800	6.71166000	0.00000000
H	-1.30890100	2.95006900	0.00000000
H	-2.25107600	7.73951100	0.00000000
C	-1.43751300	3.88945600	2.51269100
O	-1.65288900	4.43407200	3.60626300
N	-1.11445300	2.58077000	2.43816600
H	-1.15175100	2.07018600	3.30646200
H	-0.95531400	2.05011800	1.57974500
C	-1.43751300	3.88945600	-2.51269100
O	-1.65288900	4.43407200	-3.60626300
N	-1.11445300	2.58077000	-2.43816600
H	-1.15175100	2.07018600	-3.30646200
H	-0.95531400	2.05011800	-1.57974500
F	-0.62380500	1.19558400	0.00000000
N	-1.84001300	6.77882500	2.41854900
C	-1.62194300	8.10147300	2.59417800
H	-1.86056700	6.16509100	3.24423700
O	-1.40265200	8.94248000	1.72328400
H	-1.65960700	8.38586800	3.65478200
N	-1.84001300	6.77882500	-2.41854900
C	-1.62194300	8.10147300	-2.59417800
H	-1.86056700	6.16509100	-3.24423700
O	-1.40265200	8.94248000	-1.72328400
H	-1.65960700	8.38586800	-3.65478200
Li	-0.51900100	9.22169100	0.00000000
C	1.77182200	-6.05787800	1.21630900
C	1.53757200	-4.66339700	1.23023100

C	1.45711900	-4.01670200	0.00000000
C	1.53757200	-4.66339700	-1.23023100
C	1.77182200	-6.05787800	-1.21630900
C	1.94903800	-6.71166000	0.00000000
H	1.30890100	-2.95006900	0.00000000
H	2.25107600	-7.73951100	0.00000000
C	1.43751300	-3.88945600	2.51269100
O	1.65288900	-4.43407200	3.60626300
N	1.11445300	-2.58077000	2.43816600
H	1.15175100	-2.07018600	3.30646200
H	0.95531400	-2.05011800	1.57974500
C	1.43751300	-3.88945600	-2.51269100
O	1.65288900	-4.43407200	-3.60626300
N	1.11445300	-2.58077000	-2.43816600
H	1.15175100	-2.07018600	-3.30646200
H	0.95531400	-2.05011800	-1.57974500
F	0.62380500	-1.19558400	0.00000000
N	1.84001300	-6.77882500	2.41854900
C	1.62194300	-8.10147300	2.59417800
H	1.86056700	-6.16509100	3.24423700
O	1.40265200	-8.94248000	1.72328400
H	1.65960700	-8.38586800	3.65478200
N	1.84001300	-6.77882500	-2.41854900
C	1.62194300	-8.10147300	-2.59417800
H	1.86056700	-6.16509100	-3.24423700
O	1.40265200	-8.94248000	-1.72328400
H	1.65960700	-8.38586800	-3.65478200
Li	0.51900100	-9.22169100	0.00000000
C	-2.16525200	-3.95158700	1.21671100
C	-1.94504300	-5.34972100	1.22950600
C	-1.86526900	-5.99574500	0.00000000
C	-1.94504300	-5.34972100	-1.22950600

C	-2.16525200	-3.95158700	-1.21671100
C	-2.32247300	-3.29218900	0.00000000
H	-1.72250700	-7.06418300	0.00000000
H	-2.59463000	-2.25691900	0.00000000
C	-1.84795200	-6.13076600	2.50693000
O	-2.00882200	-5.58133900	3.60854700
N	-1.57965400	-7.45226500	2.42379600
H	-1.60904800	-7.95286900	3.29876100
H	-1.48350200	-7.99795900	1.56019100
C	-1.84795200	-6.13076600	-2.50693000
O	-2.00882200	-5.58133900	-3.60854700
N	-1.57965400	-7.45226500	-2.42379600
H	-1.60904800	-7.95286900	-3.29876100
H	-1.48350200	-7.99795900	-1.56019100
F	-1.16125700	-8.84607600	0.00000000
N	-2.24302300	-3.24446100	2.42475300
C	-2.07170400	-1.91783900	2.62573100
H	-2.24240500	-3.86988500	3.24258100
O	-1.88808800	-1.04771800	1.77845900
H	-2.11311400	-1.66443800	3.69539500
N	-2.24302300	-3.24446100	-2.42475300
C	-2.07170400	-1.91783900	-2.62573100
H	-2.24240500	-3.86988500	-3.24258100
O	-1.88808800	-1.04771800	-1.77845900
H	-2.11311400	-1.66443800	-3.69539500
Li	-1.09665300	-0.55145800	0.00000000

M052X/6-31+G(d) Optimized [(m-LiF)$_2$]$_2$-B Complex

C	0.02792900	6.35261700	1.23465100
C	-0.12053800	4.95227800	1.28042000
C	-0.25896500	4.27988200	0.07112600
C	-0.20317100	4.90341700	-1.17045500
C	-0.05189400	6.30380000	-1.19063200
C	0.00015700	7.01053900	0.00788600
H	-0.41485200	3.21467300	0.09761300
H	-0.03234100	8.08281000	-0.01306700
C	-0.19599100	4.20260000	2.57693300
O	-0.26403200	4.80199600	3.65974700
N	-0.19962400	2.85899000	2.52516900
H	-0.36172500	2.39355100	3.40409200
H	-0.20362600	2.28954700	1.67859100
C	-0.36929200	4.10362700	-2.42791900
O	-0.51578800	4.66111300	-3.52545700
N	-0.36798200	2.76338000	-2.32301400
H	-0.60219800	2.26242000	-3.16521900
H	-0.31344100	2.22754800	-1.45648800
F	-0.21101100	1.32213700	0.12679900
N	0.17353600	7.09012700	2.41717000
C	0.76493000	8.29348000	2.54451500
H	-0.01130100	6.54058500	3.26069900
O	1.20746800	9.00000300	1.63834600
H	0.82559300	8.62166100	3.58635100
N	0.01727800	6.99354300	-2.40883500
C	0.60219900	8.18788100	-2.62094900
H	-0.22513400	6.41252000	-3.21602000
O	1.10351000	8.92738100	-1.77354100
H	0.59692600	8.47476700	-3.67670700
Li	2.07452600	8.86626700	-0.09544100

C	3.27219400	3.05418500	1.18976600
C	3.43929300	4.45417700	1.17149100
C	3.49756200	5.07927900	-0.06818500
C	3.35482200	4.41100400	-1.27834800
C	3.18752600	3.01144500	-1.23583000
C	3.19687500	2.35041700	-0.00994500
H	3.65830400	6.14445200	-0.09238400
H	3.19408600	1.27861000	0.00927000
C	3.60572800	5.25812000	2.42563100
O	3.69780200	4.70862300	3.53392300
N	3.65519700	6.59800200	2.31136600
H	3.87009800	7.08928800	3.16501600
H	3.65828600	7.13174900	1.43727200
C	3.43525500	5.17045700	-2.56840900
O	3.44637300	4.58275500	-3.66070400
N	3.49870100	6.51294500	-2.50510000
H	3.65515900	6.97323000	-3.38823800
H	3.56197400	7.07704100	-1.65272200
F	3.55221600	8.01596100	-0.12225000
N	3.21986500	2.37139400	2.41102000
C	2.67508100	1.16177500	2.64813300
H	3.44997200	2.96717400	3.21143700
O	2.18819500	0.38804700	1.82638700
H	2.70257900	0.90088300	3.71147800
N	3.04924600	2.28753500	-2.42620900
C	2.48794600	1.07275300	-2.58377400
H	3.22255100	2.85472700	-3.26086200
O	2.06032400	0.32669600	-1.70500600
H	2.43939100	0.77837900	-3.63768800
Li	1.20746800	0.18586100	0.10193700
C	-3.27219400	-3.05418500	1.18976600
C	-3.43929300	-4.45417700	1.17149100

C	-3.49756200	-5.07927900	-0.06818500
C	-3.35482200	-4.41100400	-1.27834800
C	-3.18752600	-3.01144500	-1.23583000
C	-3.19687500	-2.35041700	-0.00994500
H	-3.65830400	-6.14445200	-0.09238400
H	-3.19408600	-1.27861000	0.00927000
C	-3.60572800	-5.25812000	2.42563100
O	-3.69780200	-4.70862300	3.53392300
N	-3.65519700	-6.59800200	2.31136600
H	-3.87009800	-7.08928800	3.16501600
H	-3.65828600	-7.13174900	1.43727200
C	-3.43525500	-5.17045700	-2.56840900
O	-3.44637300	-4.58275500	-3.66070400
N	-3.49870100	-6.51294500	-2.50510000
H	-3.65515900	-6.97323000	-3.38823800
H	-3.56197400	-7.07704100	-1.65272200
F	-3.55221600	-8.01596100	-0.12225000
N	-3.21986500	-2.37139400	2.41102000
C	-2.67508100	-1.16177500	2.64813300
H	-3.44997200	-2.96717400	3.21143700
O	-2.18819500	-0.38804700	1.82638700
H	-2.70257900	-0.90088300	3.71147800
N	-3.04924600	-2.28753500	-2.42620900
C	-2.48794600	-1.07275300	-2.58377400
H	-3.22255100	-2.85472700	-3.26086200
O	-2.06032400	-0.32669600	-1.70500600
H	-2.43939100	-0.77837900	-3.63768800
Li	-1.20746800	-0.18586100	0.10193700
C	-0.02792900	-6.35261700	1.23465100
C	0.12053800	-4.95227800	1.28042000
C	0.25896500	-4.27988200	0.07112600
C	0.20317100	-4.90341700	-1.17045500

C	0.05189400	-6.30380000	-1.19063200
C	-0.00015700	-7.01053900	0.00788600
H	0.41485200	-3.21467300	0.09761300
H	0.03234100	-8.08281000	-0.01306700
C	0.19599100	-4.20260000	2.57693300
O	0.26403200	-4.80199600	3.65974700
N	0.19962400	-2.85899000	2.52516900
H	0.36172500	-2.39355100	3.40409200
H	0.20362600	-2.28954700	1.67859100
C	0.36929200	-4.10362700	-2.42791900
O	0.51578800	-4.66111300	-3.52545700
N	0.36798200	-2.76338000	-2.32301400
H	0.60219800	-2.26242000	-3.16521900
H	0.31344100	-2.22754800	-1.45648800
F	0.21101100	-1.32213700	0.12679900
N	-0.17353600	-7.09012700	2.41717000
C	-0.76493000	-8.29348000	2.54451500
H	0.01130100	-6.54058500	3.26069900
O	-1.20746800	-9.00000300	1.63834600
H	-0.82559300	-8.62166100	3.58635100
N	-0.01727800	-6.99354300	-2.40883500
C	-0.60219900	-8.18788100	-2.62094900
H	0.22513400	-6.41252000	-3.21602000
O	-1.10351000	-8.92738100	-1.77354100
H	-0.59692600	-8.47476700	-3.67670700
Li	-2.07452600	-8.86626700	-0.09544100

wB97XD/6-311+G(d,p) Optimized [(m-LiF)$_2$]$_2$-B Complex

C	2.16627400	3.86476000	1.21240400
C	1.93455400	5.25385100	1.22392400
C	1.84339300	5.89817300	0.00000000
C	1.93455400	5.25385100	-1.22392400
C	2.16627400	3.86476000	-1.21240400
C	2.31767100	3.20224300	0.00000000
H	1.68921600	6.96677100	0.00000000
H	2.60659700	2.16970700	0.00000000
C	1.84376500	6.04009200	2.49815000
O	2.02438300	5.50363300	3.59547300
N	1.56318500	7.35384700	2.40775300
H	1.60364800	7.85843800	3.27835900
H	1.46964500	7.89546000	1.54144300
C	1.84376500	6.04009200	-2.49815000
O	2.02438300	5.50363300	-3.59547300
N	1.56318500	7.35384700	-2.40775300
H	1.60364800	7.85843800	-3.27835900
H	1.46964500	7.89546000	-1.54144300
F	1.19029900	8.74751200	0.00000000
N	2.27457700	3.17260300	2.42331300
C	2.12011500	1.85132700	2.63724700
H	2.27774500	3.80347800	3.23385100
O	1.93358300	0.97663900	1.80391200
H	2.18218300	1.60575900	3.70747100
N	2.27457700	3.17260300	-2.42331300
C	2.12011500	1.85132700	-2.63724700
H	2.27774500	3.80347800	-3.23385100
O	1.93358300	0.97663900	-1.80391200
H	2.18218300	1.60575900	-3.70747100
Li	1.10609000	0.52735800	0.00000000

C	-1.71420300	6.08870700	1.21235900
C	-1.47363300	4.70241000	1.22472200
C	-1.38440300	4.05746400	0.00000000
C	-1.47363300	4.70241000	-1.22472200
C	-1.71420300	6.08870700	-1.21235900
C	-1.88232900	6.74598000	0.00000000
H	-1.22949300	2.99016200	0.00000000
H	-2.20162800	7.77037300	0.00000000
C	-1.38484000	3.92221000	2.50357100
O	-1.62242800	4.45295100	3.59178300
N	-1.05150100	2.62129100	2.42241400
H	-1.10323900	2.10539700	3.28519600
H	-0.89053000	2.09527000	1.56155700
C	-1.38484000	3.92221000	-2.50357100
O	-1.62242800	4.45295100	-3.59178300
N	-1.05150100	2.62129100	-2.42241400
H	-1.10323900	2.10539700	-3.28519600
H	-0.89053000	2.09527000	-1.56155700
F	-0.59504200	1.22892600	0.00000000
N	-1.81422200	6.79336200	2.41800200
C	-1.61969000	8.11229100	2.60821500
H	-1.84017700	6.17257200	3.23539800
O	-1.40151500	8.96026300	1.75234400
H	-1.67900400	8.38791900	3.66987900
N	-1.81422200	6.79336200	-2.41800200
C	-1.61969000	8.11229100	-2.60821500
H	-1.84017700	6.17257200	-3.23539800
O	-1.40151500	8.96026300	-1.75234400
H	-1.67900400	8.38791900	-3.66987900
Li	-0.49387700	9.16628700	0.00000000
C	1.71420300	-6.08870700	1.21235900
C	1.47363300	-4.70241000	1.22472200

C	1.38440300	-4.05746400	0.00000000
C	1.47363300	-4.70241000	-1.22472200
C	1.71420300	-6.08870700	-1.21235900
C	1.88232900	-6.74598000	0.00000000
H	1.22949300	-2.99016200	0.00000000
H	2.20162800	-7.77037300	0.00000000
C	1.38484000	-3.92221000	2.50357100
O	1.62242800	-4.45295100	3.59178300
N	1.05150100	-2.62129100	2.42241400
H	1.10323900	-2.10539700	3.28519600
H	0.89053000	-2.09527000	1.56155700
C	1.38484000	-3.92221000	-2.50357100
O	1.62242800	-4.45295100	-3.59178300
N	1.05150100	-2.62129100	-2.42241400
H	1.10323900	-2.10539700	-3.28519600
H	0.89053000	-2.09527000	-1.56155700
F	0.59504200	-1.22892600	0.00000000
N	1.81422200	-6.79336200	2.41800200
C	1.61969000	-8.11229100	2.60821500
H	1.84017700	-6.17257200	3.23539800
O	1.40151500	-8.96026300	1.75234400
H	1.67900400	-8.38791900	3.66987900
N	1.81422200	-6.79336200	-2.41800200
C	1.61969000	-8.11229100	-2.60821500
H	1.84017700	-6.17257200	-3.23539800
O	1.40151500	-8.96026300	-1.75234400
H	1.67900400	-8.38791900	-3.66987900
Li	0.49387700	-9.16628700	0.00000000
C	-2.16627400	-3.86476000	1.21240400
C	-1.93455400	-5.25385100	1.22392400
C	-1.84339300	-5.89817300	0.00000000
C	-1.93455400	-5.25385100	-1.22392400

C	-2.16627400	-3.86476000	-1.21240400
C	-2.31767100	-3.20224300	0.00000000
H	-1.68921600	-6.96677100	0.00000000
H	-2.60659700	-2.16970700	0.00000000
C	-1.84376500	-6.04009200	2.49815000
O	-2.02438300	-5.50363300	3.59547300
N	-1.56318500	-7.35384700	2.40775300
H	-1.60364800	-7.85843800	3.27835900
H	-1.46964500	-7.89546000	1.54144300
C	-1.84376500	-6.04009200	-2.49815000
O	-2.02438300	-5.50363300	-3.59547300
N	-1.56318500	-7.35384700	-2.40775300
H	-1.60364800	-7.85843800	-3.27835900
H	-1.46964500	-7.89546000	-1.54144300
F	-1.19029900	-8.74751200	0.00000000
N	-2.27457700	-3.17260300	2.42331300
C	-2.12011500	-1.85132700	2.63724700
H	-2.27774500	-3.80347800	3.23385100
O	-1.93358300	-0.97663900	1.80391200
H	-2.18218300	-1.60575900	3.70747100
N	-2.27457700	-3.17260300	-2.42331300
C	-2.12011500	-1.85132700	-2.63724700
H	-2.27774500	-3.80347800	-3.23385100
O	-1.93358300	-0.97663900	-1.80391200
H	-2.18218300	-1.60575900	-3.70747100
Li	-1.10609000	-0.52735800	0.00000000

B3LYP/6-31+G(d) Optimized [(m-LiF)$_2$]$_4$-B Complex

C	2.57033300	14.20613500	1.21543700
C	2.48575400	15.61902700	1.22927800
C	2.50161500	16.27874700	0.00000000
C	2.48575400	15.61902700	-1.22927800
C	2.57033300	14.20613500	-1.21543700
C	2.66443600	13.52644900	0.00000000
H	2.49325800	17.35779400	0.00000000
H	2.81010000	12.46230200	0.00000000
C	2.35441600	16.39692900	2.50452000
O	2.65604700	15.89205600	3.60500100
N	1.88622500	17.65962900	2.40063700
H	1.82048000	18.18501900	3.26297800
H	1.49169100	18.07336400	1.54114500
C	2.35441600	16.39692900	-2.50452000
O	2.65604700	15.89205600	-3.60500100
N	1.88622500	17.65962900	-2.40063700
H	1.82048000	18.18501900	-3.26297800
H	1.49169100	18.07336400	-1.54114500
F	0.79894600	18.65527500	0.00000000
N	2.56572000	13.49507100	2.42818300
C	2.22653500	12.19849800	2.62613000
H	2.69939000	14.10411900	3.24684500
O	1.90605800	11.36739000	1.77172900
H	2.26228400	11.92568100	3.69238200
N	2.56572000	13.49507100	-2.42818300
C	2.22653500	12.19849800	-2.62613000
H	2.69939000	14.10411900	-3.24684500
O	1.90605800	11.36739000	-1.77172900
H	2.26228400	11.92568100	-3.69238200
Li	1.06446700	10.77112500	0.00000000

C	-2.50841000	15.67219000	1.21616400
C	-2.33864000	14.26742300	1.23089400
C	-2.30713800	13.60909400	0.00000000
C	-2.33864000	14.26742300	-1.23089400
C	-2.50841000	15.67219000	-1.21616400
C	-2.65334800	16.34071900	0.00000000
H	-2.22590800	12.53249900	0.00000000
H	-2.89115700	17.38931800	0.00000000
C	-2.19973900	13.50130000	2.51341900
O	-2.51662900	14.01269800	3.60603600
N	-1.71942200	12.24255100	2.43214900
H	-1.66860300	11.72192400	3.29763800
H	-1.35882600	11.79465500	1.58170900
C	-2.19973900	13.50130000	-2.51341900
O	-2.51662900	14.01269800	-3.60603600
N	-1.71942200	12.24255100	-2.43214900
H	-1.66860300	11.72192400	-3.29763800
H	-1.35882600	11.79465500	-1.58170900
F	-0.76183500	11.05101400	0.00000000
N	-2.53620500	16.39827000	2.42078100
C	-2.20089000	17.69996100	2.59090000
H	-2.64834100	15.79695400	3.24834600
O	-1.86676400	18.50195000	1.71191300
H	-2.25195500	18.01023700	3.64470700
N	-2.53620500	16.39827000	-2.42078100
C	-2.20089000	17.69996100	-2.59090000
H	-2.64834100	15.79695400	-3.24834600
O	-1.86676400	18.50195000	-1.71191300
H	-2.25195500	18.01023700	-3.64470700
Li	-0.91646900	18.71461800	0.00000000
C	2.45088400	4.25264200	1.21622500
C	2.37928600	5.66737100	1.23077600

C	2.39071700	6.32564300	0.00000000
C	2.37928600	5.66737100	-1.23077600
C	2.45088400	4.25264200	-1.21622500
C	2.53474200	3.57385000	0.00000000
H	2.38666400	7.40497300	0.00000000
H	2.66744500	2.50852600	0.00000000
C	2.28882100	6.44515900	2.50971700
O	2.56984400	5.92203700	3.60703000
N	1.88835800	7.73201500	2.42257400
H	1.86710300	8.25734500	3.28638700
H	1.53939700	8.19128800	1.57351200
C	2.28882100	6.44515900	-2.50971700
O	2.56984400	5.92203700	-3.60703000
N	1.88835800	7.73201500	-2.42257400
H	1.86710300	8.25734500	-3.28638700
H	1.53939700	8.19128800	-1.57351200
F	0.94066500	8.94224900	0.00000000
N	2.44535200	3.53796700	2.42581100
C	2.12011600	2.23631600	2.62005300
H	2.57711600	4.14455000	3.24662300
O	1.81379100	1.40268900	1.76361400
H	2.15392300	1.96314100	3.68623000
N	2.44535200	3.53796700	-2.42581100
C	2.12011600	2.23631600	-2.62005300
H	2.57711600	4.14455000	-3.24662300
O	1.81379100	1.40268900	-1.76361400
H	2.15392300	1.96314100	-3.68623000
Li	0.96886900	0.77783400	0.00000000
C	-2.35640400	5.74731800	1.21625600
C	-2.28357900	4.33260100	1.23082900
C	-2.29419200	3.67440000	0.00000000
C	-2.28357900	4.33260100	-1.23082900

C	-2.35640400	5.74731800	-1.21625600
C	-2.44087200	6.42595200	0.00000000
H	-2.28876000	2.59505200	0.00000000
H	-2.57455100	7.49113800	0.00000000
C	-2.19283600	3.55482900	2.50978700
O	-2.47211800	4.07845400	3.60730000
N	-1.79425500	2.26733900	2.42279500
H	-1.77105800	1.74326800	3.28734900
H	-1.44679400	1.80711300	1.57364600
C	-2.19283600	3.55482900	-2.50978700
O	-2.47211800	4.07845400	-3.60730000
N	-1.79425500	2.26733900	-2.42279500
H	-1.77105800	1.74326800	-3.28734900
H	-1.44679400	1.80711300	-1.57364600
F	-0.85444300	1.05037300	0.00000000
N	-2.35146100	6.46199500	2.42581500
C	-2.02908400	7.76430100	2.62028700
H	-2.48133700	5.85485600	3.24656100
O	-1.72551900	8.59923900	1.76409900
H	-2.06261700	8.03677500	3.68665300
N	-2.35146100	6.46199500	-2.42581500
C	-2.02908400	7.76430100	-2.62028700
H	-2.48133700	5.85485600	-3.24656100
O	-1.72551900	8.59923900	-1.76409900
H	-2.06261700	8.03677500	-3.68665300
Li	-0.87852500	9.22018200	0.00000000
C	2.35640400	-5.74731800	1.21625600
C	2.28357900	-4.33260100	1.23082900
C	2.29419200	-3.67440000	0.00000000
C	2.28357900	-4.33260100	-1.23082900
C	2.35640400	-5.74731800	-1.21625600
C	2.44087200	-6.42595200	0.00000000

H	2.28876000	-2.59505200	0.00000000
H	2.57455100	-7.49113800	0.00000000
C	2.19283600	-3.55482900	2.50978700
O	2.47211800	-4.07845400	3.60730000
N	1.79425500	-2.26733900	2.42279500
H	1.77105800	-1.74326800	3.28734900
H	1.44679400	-1.80711300	1.57364600
C	2.19283600	-3.55482900	-2.50978700
O	2.47211800	-4.07845400	-3.60730000
N	1.79425500	-2.26733900	-2.42279500
H	1.77105800	-1.74326800	-3.28734900
H	1.44679400	-1.80711300	-1.57364600
F	0.85444300	-1.05037300	0.00000000
N	2.35146100	-6.46199500	2.42581500
C	2.02908400	-7.76430100	2.62028700
H	2.48133700	-5.85485600	3.24656100
O	1.72551900	-8.59923900	1.76409900
H	2.06261700	-8.03677500	3.68665300
N	2.35146100	-6.46199500	-2.42581500
C	2.02908400	-7.76430100	-2.62028700
H	2.48133700	-5.85485600	-3.24656100
O	1.72551900	-8.59923900	-1.76409900
H	2.06261700	-8.03677500	-3.68665300
Li	0.87852500	-9.22018200	0.00000000
C	-2.45088400	-4.25264200	1.21622500
C	-2.37928600	-5.66737100	1.23077600
C	-2.39071700	-6.32564300	0.00000000
C	-2.37928600	-5.66737100	-1.23077600
C	-2.45088400	-4.25264200	-1.21622500
C	-2.53474200	-3.57385000	0.00000000
H	-2.38666400	-7.40497300	0.00000000
H	-2.66744500	-2.50852600	0.00000000

C	-2.28882100	-6.44515900	2.50971700
O	-2.56984400	-5.92203700	3.60703000
N	-1.88835800	-7.73201500	2.42257400
H	-1.86710300	-8.25734500	3.28638700
H	-1.53939700	-8.19128800	1.57351200
C	-2.28882100	-6.44515900	-2.50971700
O	-2.56984400	-5.92203700	-3.60703000
N	-1.88835800	-7.73201500	-2.42257400
H	-1.86710300	-8.25734500	-3.28638700
H	-1.53939700	-8.19128800	-1.57351200
F	-0.94066500	-8.94224900	0.00000000
N	-2.44535200	-3.53796700	2.42581100
C	-2.12011600	-2.23631600	2.62005300
H	-2.57711600	-4.14455000	3.24662300
O	-1.81379100	-1.40268900	1.76361400
H	-2.15392300	-1.96314100	3.68623000
N	-2.44535200	-3.53796700	-2.42581100
C	-2.12011600	-2.23631600	-2.62005300
H	-2.57711600	-4.14455000	-3.24662300
O	-1.81379100	-1.40268900	-1.76361400
H	-2.15392300	-1.96314100	-3.68623000
Li	-0.96886900	-0.77783400	0.00000000
C	2.50841000	-15.67219000	1.21616400
C	2.33864000	-14.26742300	1.23089400
C	2.30713800	-13.60909400	0.00000000
C	2.33864000	-14.26742300	-1.23089400
C	2.50841000	-15.67219000	-1.21616400
C	2.65334800	-16.34071900	0.00000000
H	2.22590800	-12.53249900	0.00000000
H	2.89115700	-17.38931800	0.00000000
C	2.19973900	-13.50130000	2.51341900
O	2.51662900	-14.01269800	3.60603600

N	1.71942200	-12.24255100	2.43214900
H	1.66860300	-11.72192400	3.29763800
H	1.35882600	-11.79465500	1.58170900
C	2.19973900	-13.50130000	-2.51341900
O	2.51662900	-14.01269800	-3.60603600
N	1.71942200	-12.24255100	-2.43214900
H	1.66860300	-11.72192400	-3.29763800
H	1.35882600	-11.79465500	-1.58170900
F	0.76183500	-11.05101400	0.00000000
N	2.53620500	-16.39827000	2.42078100
C	2.20089000	-17.69996100	2.59090000
H	2.64834100	-15.79695400	3.24834600
O	1.86676400	-18.50195000	1.71191300
H	2.25195500	-18.01023700	3.64470700
N	2.53620500	-16.39827000	-2.42078100
C	2.20089000	-17.69996100	-2.59090000
H	2.64834100	-15.79695400	-3.24834600
O	1.86676400	-18.50195000	-1.71191300
H	2.25195500	-18.01023700	-3.64470700
Li	0.91646900	-18.71461800	0.00000000
C	-2.57033300	-14.20613500	1.21543700
C	-2.48575400	-15.61902700	1.22927800
C	-2.50161500	-16.27874700	0.00000000
C	-2.48575400	-15.61902700	-1.22927800
C	-2.57033300	-14.20613500	-1.21543700
C	-2.66443600	-13.52644900	0.00000000
H	-2.49325800	-17.35779400	0.00000000
H	-2.81010000	-12.46230200	0.00000000
C	-2.35441600	-16.39692900	2.50452000
O	-2.65604700	-15.89205600	3.60500100
N	-1.88622500	-17.65962900	2.40063700
H	-1.82048000	-18.18501900	3.26297800

H	-1.49169100	-18.07336400	1.54114500
C	-2.35441600	-16.39692900	-2.50452000
O	-2.65604700	-15.89205600	-3.60500100
N	-1.88622500	-17.65962900	-2.40063700
H	-1.82048000	-18.18501900	-3.26297800
H	-1.49169100	-18.07336400	-1.54114500
F	-0.79894600	-18.65527500	0.00000000
N	-2.56572000	-13.49507100	2.42818300
C	-2.22653500	-12.19849800	2.62613000
H	-2.69939000	-14.10411900	3.24684500
O	-1.90605800	-11.36739000	1.77172900
H	-2.26228400	-11.92568100	3.69238200
N	-2.56572000	-13.49507100	-2.42818300
C	-2.22653500	-12.19849800	-2.62613000
H	-2.69939000	-14.10411900	-3.24684500
O	-1.90605800	-11.36739000	-1.77172900
H	-2.26228400	-11.92568100	-3.69238200
Li	-1.06446700	-10.77112500	0.00000000

M052X/6-31+G(d) Optimized [(m-LiF)$_2$]$_4$-B Complex

C	2.12176900	3.95887700	1.21357100
C	1.87712700	5.34770600	1.22609100
C	1.78829200	5.99647700	0.00000000
C	1.87712700	5.34770600	-1.22609100
C	2.12176900	3.95887700	-1.21357100
C	2.28520700	3.29579100	0.00000000
H	1.64597900	7.06359500	0.00000000
H	2.59102100	2.26886600	0.00000000
C	1.77247400	6.12959200	2.50093800
O	2.03532000	5.61152200	3.59665600
N	1.39130300	7.41671300	2.41880600
H	1.43988400	7.93775900	3.27982600
H	1.19863400	7.93578900	1.56188100
C	1.77247400	6.12959200	-2.50093800
O	2.03532000	5.61152200	-3.59665600
N	1.39130300	7.41671300	-2.41880600
H	1.43988400	7.93775900	-3.27982600
H	1.19863400	7.93578900	-1.56188100
F	0.85891800	8.80732400	0.00000000
N	2.23494300	3.25811800	2.42015800
C	2.07286300	1.93460200	2.61774800
H	2.27115500	3.87231600	3.23821500
O	1.86923800	1.07149300	1.76683600
H	2.14358200	1.66759800	3.67756300
N	2.23494300	3.25811800	-2.42015800
C	2.07286300	1.93460200	-2.61774800
H	2.27115500	3.87231600	-3.23821500
O	1.86923800	1.07149300	-1.76683600
H	2.14358200	1.66759800	-3.67756300
Li	1.06886600	0.58876300	0.00000000

C	-1.90376600	6.02085700	1.21361500
C	-1.65366000	4.63297700	1.22612900
C	-1.56123700	3.98486700	0.00000000
C	-1.65366000	4.63297700	-1.22612900
C	-1.90376600	6.02085700	-1.21361500
C	-2.07006000	6.68323400	0.00000000
H	-1.41256000	2.91861700	0.00000000
H	-2.38023400	7.70888900	0.00000000
C	-1.54723600	3.85122200	2.50095500
O	-1.80593400	4.37027000	3.59725900
N	-1.16983300	2.56302800	2.41873800
H	-1.21503200	2.04370700	3.28098700
H	-0.98164500	2.04200300	1.56199000
C	-1.54723600	3.85122200	-2.50095500
O	-1.80593400	4.37027000	-3.59725900
N	-1.16983300	2.56302800	-2.41873800
H	-1.21503200	2.04370700	-3.28098700
H	-0.98164500	2.04200300	-1.56199000
F	-0.64988500	1.16841400	0.00000000
N	-2.01906900	6.72132800	2.42018000
C	-1.86141300	8.04531000	2.61775800
H	-2.05074100	6.10696000	3.23837100
O	-1.66187200	8.90937000	1.76674000
H	-1.93188800	8.31189200	3.67768800
N	-2.01906900	6.72132800	-2.42018000
C	-1.86141300	8.04531000	-2.61775800
H	-2.05074100	6.10696000	-3.23837100
O	-1.66187200	8.90937000	-1.76674000
H	-1.93188800	8.31189200	-3.67768800
Li	-0.85856100	9.38620300	0.00000000
C	2.20757900	13.93241200	1.21385400
C	1.89096100	15.30647400	1.22592600

C	1.76708300	15.94932000	0.00000000
C	1.89096100	15.30647400	-1.22592600
C	2.20757900	13.93241200	-1.21385400
C	2.40740400	13.27898700	0.00000000
H	1.55862700	17.00650400	0.00000000
H	2.76834500	12.26963900	0.00000000
C	1.74107300	16.08394800	2.49881900
O	1.98670600	15.56817300	3.59987700
N	1.33388100	17.36327200	2.40953000
H	1.34718800	17.87506500	3.27794100
H	1.17511400	17.88942200	1.54526500
C	1.74107300	16.08394800	-2.49881900
O	1.98670600	15.56817300	-3.59987700
N	1.33388100	17.36327200	-2.40953000
H	1.34718800	17.87506500	-3.27794100
H	1.17511400	17.88942200	-1.54526500
F	0.81120900	18.72590400	0.00000000
N	2.35628200	13.23872600	2.42090700
C	2.24917500	11.91008200	2.61908600
H	2.35000700	13.85471700	3.23885100
O	2.07782800	11.03928400	1.76853700
H	2.33382600	11.64584200	3.67863100
N	2.35628200	13.23872600	-2.42090700
C	2.24917500	11.91008200	-2.61908600
H	2.35000700	13.85471700	-3.23885100
O	2.07782800	11.03928400	-1.76853700
H	2.33382600	11.64584200	-3.67863100
Li	1.27788800	10.56491900	0.00000000
C	-1.96360600	15.91542000	1.21356200
C	-1.62957600	14.54681300	1.22637700
C	-1.50212500	13.90392800	0.00000000
C	-1.62957600	14.54681300	-1.22637700

C	-1.96360600	15.91542000	-1.21356200
C	-2.18425100	16.56139700	0.00000000
H	-1.28923100	12.84830600	0.00000000
H	-2.57948200	17.55884200	0.00000000
C	-1.47780200	13.77727000	2.50440300
O	-1.77443000	14.28500100	3.59576200
N	-1.02176700	12.51497700	2.42610100
H	-1.03938900	11.99456800	3.28871800
H	-0.80754900	12.00372600	1.56938700
C	-1.47780200	13.77727000	-2.50440300
O	-1.77443000	14.28500100	-3.59576200
N	-1.02176700	12.51497700	-2.42610100
H	-1.03938900	11.99456800	-3.28871800
H	-0.80754900	12.00372600	-1.56938700
F	-0.44359700	11.14469500	0.00000000
N	-2.10394600	16.62309200	2.41505500
C	-1.95781100	17.95102200	2.58531100
H	-2.11181000	16.01791900	3.24074700
O	-1.76099600	18.79237700	1.70803400
H	-2.03464300	18.24920000	3.63510400
N	-2.10394600	16.62309200	-2.41505500
C	-1.95781100	17.95102200	-2.58531100
H	-2.11181000	16.01791900	-3.24074700
O	-1.76099600	18.79237700	-1.70803400
H	-2.03464300	18.24920000	-3.63510400
Li	-0.86936000	19.01959400	0.00000000
C	1.96360600	-15.91542000	1.21356200
C	1.62957600	-14.54681300	1.22637700
C	1.50212500	-13.90392800	0.00000000
C	1.62957600	-14.54681300	-1.22637700
C	1.96360600	-15.91542000	-1.21356200
C	2.18425100	-16.56139700	0.00000000

H	1.28923100	-12.84830600	0.00000000
H	2.57948200	-17.55884200	0.00000000
C	1.47780200	-13.77727000	2.50440300
O	1.77443000	-14.28500100	3.59576200
N	1.02176700	-12.51497700	2.42610100
H	1.03938900	-11.99456800	3.28871800
H	0.80754900	-12.00372600	1.56938700
C	1.47780200	-13.77727000	-2.50440300
O	1.77443000	-14.28500100	-3.59576200
N	1.02176700	-12.51497700	-2.42610100
H	1.03938900	-11.99456800	-3.28871800
H	0.80754900	-12.00372600	-1.56938700
F	0.44359700	-11.14469500	0.00000000
N	2.10394600	-16.62309200	2.41505500
C	1.95781100	-17.95102200	2.58531100
H	2.11181000	-16.01791900	3.24074700
O	1.76099600	-18.79237700	1.70803400
H	2.03464300	-18.24920000	3.63510400
N	2.10394600	-16.62309200	-2.41505500
C	1.95781100	-17.95102200	-2.58531100
H	2.11181000	-16.01791900	-3.24074700
O	1.76099600	-18.79237700	-1.70803400
H	2.03464300	-18.24920000	-3.63510400
Li	0.86936000	-19.01959400	0.00000000
C	-2.20757900	-13.93241200	1.21385400
C	-1.89096100	-15.30647400	1.22592600
C	-1.76708300	-15.94932000	0.00000000
C	-1.89096100	-15.30647400	-1.22592600
C	-2.20757900	-13.93241200	-1.21385400
C	-2.40740400	-13.27898700	0.00000000
H	-1.55862700	-17.00650400	0.00000000
H	-2.76834500	-12.26963900	0.00000000

C	-1.74107300	-16.08394800	2.49881900
O	-1.98670600	-15.56817300	3.59987700
N	-1.33388100	-17.36327200	2.40953000
H	-1.34718800	-17.87506500	3.27794100
H	-1.17511400	-17.88942200	1.54526500
C	-1.74107300	-16.08394800	-2.49881900
O	-1.98670600	-15.56817300	-3.59987700
N	-1.33388100	-17.36327200	-2.40953000
H	-1.34718800	-17.87506500	-3.27794100
H	-1.17511400	-17.88942200	-1.54526500
F	-0.81120900	-18.72590400	0.00000000
N	-2.35628200	-13.23872600	2.42090700
C	-2.24917500	-11.91008200	2.61908600
H	-2.35000700	-13.85471700	3.23885100
O	-2.07782800	-11.03928400	1.76853700
H	-2.33382600	-11.64584200	3.67863100
N	-2.35628200	-13.23872600	-2.42090700
C	-2.24917500	-11.91008200	-2.61908600
H	-2.35000700	-13.85471700	-3.23885100
O	-2.07782800	-11.03928400	-1.76853700
H	-2.33382600	-11.64584200	-3.67863100
Li	-1.27788800	-10.56491900	0.00000000
C	1.90376600	-6.02085700	1.21361500
C	1.65366000	-4.63297700	1.22612900
C	1.56123700	-3.98486700	0.00000000
C	1.65366000	-4.63297700	-1.22612900
C	1.90376600	-6.02085700	-1.21361500
C	2.07006000	-6.68323400	0.00000000
H	1.41256000	-2.91861700	0.00000000
H	2.38023400	-7.70888900	0.00000000
C	1.54723600	-3.85122200	2.50095500
O	1.80593400	-4.37027000	3.59725900

N	1.16983300	-2.56302800	2.41873800
H	1.21503200	-2.04370700	3.28098700
H	0.98164500	-2.04200300	1.56199000
C	1.54723600	-3.85122200	-2.50095500
O	1.80593400	-4.37027000	-3.59725900
N	1.16983300	-2.56302800	-2.41873800
H	1.21503200	-2.04370700	-3.28098700
H	0.98164500	-2.04200300	-1.56199000
F	0.64988500	-1.16841400	0.00000000
N	2.01906900	-6.72132800	2.42018000
C	1.86141300	-8.04531000	2.61775800
H	2.05074100	-6.10696000	3.23837100
O	1.66187200	-8.90937000	1.76674000
H	1.93188800	-8.31189200	3.67768800
N	2.01906900	-6.72132800	-2.42018000
C	1.86141300	-8.04531000	-2.61775800
H	2.05074100	-6.10696000	-3.23837100
O	1.66187200	-8.90937000	-1.76674000
H	1.93188800	-8.31189200	-3.67768800
Li	0.85856100	-9.38620300	0.00000000
C	-2.12176900	-3.95887700	1.21357100
C	-1.87712700	-5.34770600	1.22609100
C	-1.78829200	-5.99647700	0.00000000
C	-1.87712700	-5.34770600	-1.22609100
C	-2.12176900	-3.95887700	-1.21357100
C	-2.28520700	-3.29579100	0.00000000
H	-1.64597900	-7.06359500	0.00000000
H	-2.59102100	-2.26886600	0.00000000
C	-1.77247400	-6.12959200	2.50093800
O	-2.03532000	-5.61152200	3.59665600
N	-1.39130300	-7.41671300	2.41880600
H	-1.43988400	-7.93775900	3.27982600

H	-1.19863400	-7.93578900	1.56188100
C	-1.77247400	-6.12959200	-2.50093800
O	-2.03532000	-5.61152200	-3.59665600
N	-1.39130300	-7.41671300	-2.41880600
H	-1.43988400	-7.93775900	-3.27982600
H	-1.19863400	-7.93578900	-1.56188100
F	-0.85891800	-8.80732400	0.00000000
N	-2.23494300	-3.25811800	2.42015800
C	-2.07286300	-1.93460200	2.61774800
H	-2.27115500	-3.87231600	3.23821500
O	-1.86923800	-1.07149300	1.76683600
H	-2.14358200	-1.66759800	3.67756300
N	-2.23494300	-3.25811800	-2.42015800
C	-2.07286300	-1.93460200	-2.61774800
H	-2.27115500	-3.87231600	-3.23821500
O	-1.86923800	-1.07149300	-1.76683600
H	-2.14358200	-1.66759800	-3.67756300
Li	-1.06886600	-0.58876300	0.00000000
